# Overview of the Antimicrobial Compounds Produced by Members of the *Bacillus subtilis* Group

**DOI:** 10.3389/fmicb.2019.00302

**Published:** 2019-02-26

**Authors:** Simon Caulier, Catherine Nannan, Annika Gillis, Florent Licciardi, Claude Bragard, Jacques Mahillon

**Affiliations:** ^1^Laboratory of Food and Environmental Microbiology, Earth and Life Institute, Université catholique de Louvain, Louvain-la-Neuve, Belgium; ^2^Laboratory of Phytopathology-Applied Microbiology, Earth and Life Institute, Université catholique de Louvain, Louvain-la-Neuve, Belgium

**Keywords:** *Bacillus subtilis* group, bacteriocins, biocontrol, biosynthetic pathways, lipopeptides, polyketides, siderophores, volatile

## Abstract

Over the last seven decades, applications using members of the *Bacillus subtilis* group have emerged in both food processes and crop protection industries. Their ability to form survival endospores and the plethora of antimicrobial compounds they produce has generated an increased industrial interest as food preservatives, therapeutic agents and biopesticides. In the growing context of food biopreservation and biological crop protection, this review suggests a comprehensive way to visualize the antimicrobial spectrum described within the *B. subtilis* group, including volatile compounds. This classification distinguishes the bioactive metabolites based on their biosynthetic pathways and chemical nature: *i.e.*, ribosomal peptides (RPs), volatile compounds, polyketides (PKs), non-ribosomal peptides (NRPs), and hybrids between PKs and NRPs. For each clade, the chemical structure, biosynthesis and antimicrobial activity are described and exemplified. This review aims at constituting a convenient and updated classification of antimicrobial metabolites from the *B. subtilis* group, whose complex phylogeny is prone to further development.

## Introduction

The genus *Bacillus* comprises 377 species^1^ (last update in January 2019) of Gram-positive, rod-shaped bacteria (Gordon et al., 1973). Their ability to form endospores, their diversity in physiological properties, as well as their capacity to produce numerous antimicrobial compounds (AMCs) favor their ubiquitous distribution in soil, aquatic environments, food and gut microbiota of arthropods and mammals (Nicholson, 2002).

**Figure 1:**
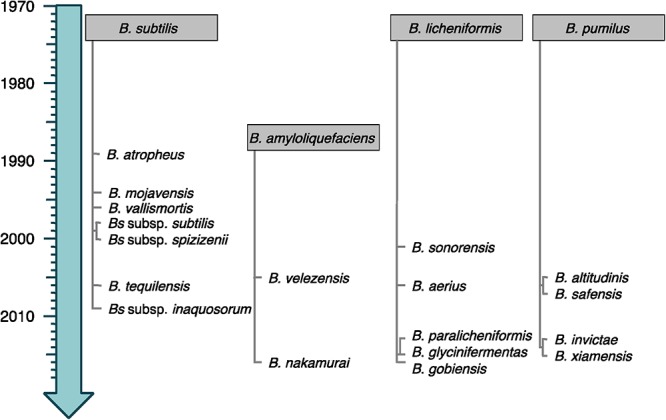
Timeline emergence of the species from the *B. subtilis* group. The species are classified following their relatedness to the closest original member of the group (gray boxes). Heterotypic synonyms are not shown.

Bacteria from the *Bacillus subtilis* group consist of small vegetative cells (<1 μm-wide) for which the strain *B. subtilis* subsp. *subtilis* 168 is considered as model organism (Barbe et al., 2009). They are usually mesophilic and neutrophilic, although some can tolerate high pH. The four original species of the group (*B. subtilis, Bacillus licheniformis*, *Bacillus pumilus*, and *Bacillus amyloliquefaciens*) were discovered more than 40 years ago (Gordon et al., 1973; Priest et al., 1987). Since then, the evolution of their molecular, chemotaxonomic and physiological characterizations led to regular re-evaluations and (re-)description of numerous novel species and subspecies (see current taxonomy of the group in Figure 1) (Fan et al., 2017).

The potential of *B. subtilis* group strains to produce a wide diversity of secondary metabolites mediating antibiosis was recognized for decades. For any given strain of the *B. subtilis* group, it is now estimated that at least 4–5% of its genome is devoted to antimicrobial compounds (AMCs) production (Stein, 2005). These molecules are mainly antimicrobial peptides (AMPs). Their structures are usually cyclic, hydrophobic and contain peculiar moieties such as D-amino acids (AA) or intramolecular thioether bonds. In addition to AMPs, volatile metabolites also constitute a large family of antimicrobials exhibiting numerous metabolic and functional roles.

**Figure 2:**
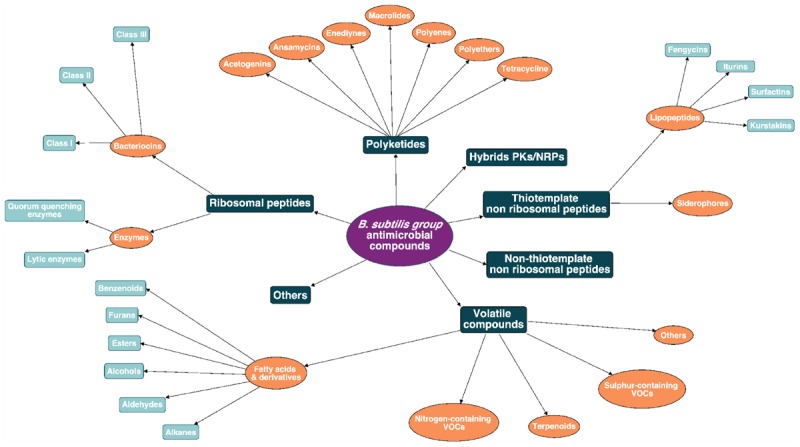
Antimicrobial molecules classes from the *B. subtilis* group. The subdivision between the classes is based on the biosynthetic pathway (*i.e.*, ribosomal peptides, polyketides, hybrids, non-ribosomal peptides, and volatile compounds).

Due to the wide diversity of these molecules, their classification is rather complex and can be based on several criteria such as their biosynthetic machinery, sources, biological functions, properties, three-dimensional structure, covalent bonding pattern or molecular targets (Tagg et al., 1976; Wang et al., 2015). Here a classification of the *B. subtilis* group antimicrobial molecules is proposed, based on their biosynthetic pathways and their chemical nature as shown in Figure 2. This review will emphasize the biosynthesis pathway and the bioactivity of the main clades of AMCs within the *B. subtilis* group: *i.e.*, the ribosomal peptides (RPs) (bacteriocins and enzymes), the polyketides (PKs), the non-ribosomal peptides (NRPs) and the volatiles. A full overview of this chart is provided as Supplementary Material (Supplementary Figure S1).

## Ribosomal Peptides

Ribosomally synthesized peptides (RPs) are usually derived from short precursors (*ca.* 100 AA) and are processed to mature compounds through post-translational modifications (Oman and van der Donk, 2009). Various enzymes mediate these modifications and therefore generate a wide diversity of chemical structures. Most of these peptides were originally referred to as “bacteriocins,” characterized as low molecular weight molecules that exhibit inhibiting growth activities against bacteria closely related to the producing strain (Klaenhammer, 1988; Chopra et al., 2015). In addition to bacteriocins, other types of enzymes exhibiting antagonistic activities are also ribosomally synthesized. However, those compounds display diverse metabolic activities such as quorum sensing (QS) mediation, cell lysis or induction of genetic competence (Schmidt, 2010; Shafi et al., 2017). It should also be noted that molecules referred to as BLIS (bacteriocins-like inhibitory substances) include AMPs for which the ribosomal synthesis has not been confirmed yet (Abriouel et al., 2011).

### *B. subtilis* Group Bacteriocins

**Figure 3:**
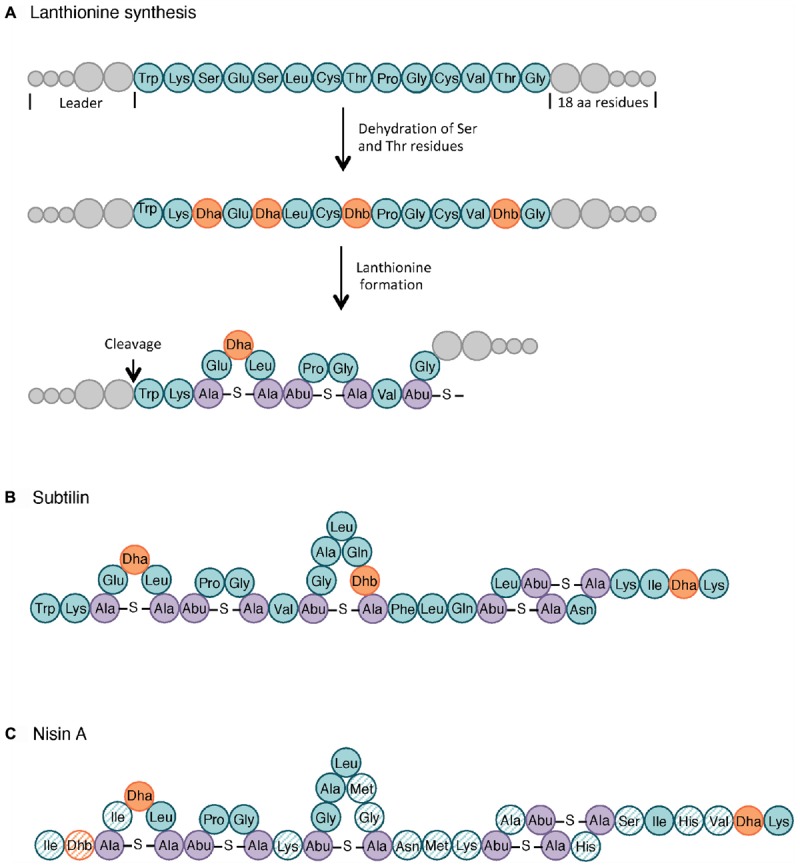
Lanthionine biosynthesis. General pathway of the lanthionine synthesis **(A)**, structure of subtilin **(B)** and nisin A **(C)**. Non-modified AA are indicated in teal whereas dehydrated serine (Dha, dehydroalanine) and threonine (Dhb, dehydrobutyrine) are colored in orange. The lanthionine (Ala-*S*-Ala, alanine-*S*-alanine) and *R*-methyllanthionine (Abu-*S*-Ala, aminobutyrate-*S*-alanine) bridges are shown in purple. The AA of nisin that differ from those in subtilin are highlighted as hatched circles. Adapted from Cotter et al. (2005) and Spieß et al. (2015).

It is estimated that 99% of the bacteria and archaea are able to produce at least one bacteriocin. Historically, lactic acid bacteria (LAB) were studied as main bacteriocin producers, mostly because of their long history of safe use in food fermentation (O’Sullivan et al., 2002). Nisin (Figure 3C), produced by *Lactobacillus lactis* subsp. *lactis*, was approved as a food additive in the 1960s and has since then been used in over 50 countries for its antimicrobial activity against Gram-positive pathogens such as *Clostridium* spp. and *Bacillus* spp. (Klaenhammer, 1988; Delves-Broughton, 1990). However, the search for new bioactive molecules has rapidly expanded to other bacteriocin-producing genera, with a particular attention, in the late 1990s, to the GRAS (generally recognized as safe) *Bacillus* species whose bacteriocin antimicrobial spectra were broader than those of LAB (Pedersen et al., 2002; Riley and Wertz, 2002; Sumi et al., 2015).

The generic biosynthetic pathway of *Bacillus* species bacteriocins includes several post-translational modifications, including the proteolytic cleavage of the leader peptide at the N-terminal end (McIntosh et al., 2009). The modifications of active peptides, its secretion and the immunity to the bacteriocin (as described below) vary depending on the bacteriocin class.

While many classifications have been suggested over the years, one reasonable way to cope with the diversity of the *Bacillus* bacteriocins is to sort them on the basis of their biosynthetic pathway as previously reported for *Streptococcus* spp. and *Enterococcus* spp. bacteriocins (Nes et al., 2007) and reviewed in Abriouel et al. (2011). Accordingly, three main classes subdivided into several subclasses can be distinguished for the *B. subtilis* group. As detailed in Table 1, Class I includes the post-translationally modified peptides such as the lantibiotics whereas the non-modified peptides are grouped in Class II; Class III involved bacteriocins larger than 10 kDa (Abriouel et al., 2011). Supplementary Table S1 summarizes the different RPs produced by the strains belonging to the *B. subtilis* group, as well as their reported antimicrobial activities.

**Table 1:** Classification of the *B. subtilis* group bacteriocins.

Class	Class description	Subclass	Subclass description
I	Post-translationally modified peptides	I.1	Single-peptide, elongated lantibiotics
		I.2	Other single-peptide lantibiotics
		I.3	Two-peptide lantibiotics
		I.4	Other modified peptides
II	Non-modified peptides	II.1	Pediocin-like peptides
		II.2	Thuricin-like peptides
		II.3	Other linear peptides
III	Large peptides (>10 kDa)		

*Adapted from Abriouel et al. (2011)*.

Class I includes small AMPs (19–38 AA) with extensive post-translational modifications. Subclasses I.1, I.2, and I.3 have in common their lantibiotic structure, which refers to inter-residual thioester bonds made of modified AA residues. As illustrated in Figure 3, lantibiotics involve 2,3-didehydroalanine (Dha) and (Z)-2,3-didehydrobutyrine (Dhb), resulting from the dehydration of serine and threonine residues, respectively. The intra-molecular addition of Dha or Dhb on a cysteine residue leads to the respective formation of lanthionine and methyllanthionine bridges (Willey and Donk, 2007). Subtilin (Figure 3B), from subclass I.1, is one of the most studied bacteriocins from the *B. subtilis* group. Its structure shares several similarities with nisin A lantibiotics, shown in Figure 3C (Guder et al., 2000; Abriouel et al., 2011). Peptides from subclass I.4 undergo other types of modifications. For instance, subtilosin A is a head-to-tail cyclic peptide with unusual inter-residue linkages (*i.e.*, Cys-Phe bond) (Marx et al., 2001; Kawulka et al., 2004).

Class II bacteriocins include small (<10 kDa), linear and non-modified peptides, resistant to heat and acido-basic treatments. They are divided in three subclasses based on a conserved AA motif near their N-terminus. The YGNGVXC (X is any AA) motif is associated to pediocin-like peptides from subclass II.1 whereas DWTXWSXL is specific to thuricin-like peptides from subclass II.2. Subclass II.3 comprises the small non-modified AMPs without any typical motif in their AA sequence (Abriouel et al., 2011). Finally, class III bacteriocins consist into large and heat labile molecules, generally characterized by a phospholipase activity (Cleveland et al., 2001).

Because of their wide diversity, bacteriocins display different modes of action such as protoplasm vesicularization, pore formation or cell disintegration (Sumi et al., 2015). They are generally bactericidal with some exceptions that exhibit bacteriostatic activities (Gautam and Sharma, 2009). For most class I and II bacteriocins, the target of their activity is the bacterial envelope due to their amphiphilic or hydrophobic properties. For instance, lantibiotics from subclass I.1 have a dual mode of action. On the one hand, they can inhibit the cell wall synthesis of the targeted bacteria through binding to lipid II, the major transporter of peptidoglycan subunits across the inner cell membrane. On the other hand, lipid II can be used as a docking molecule to insert the lantibiotic in the membrane leading to pore formation and ultimately to cell death as well described in Chatterjee et al. (2005) and Cotter et al. (2005). This duality has been reported for subtilin, a class I bacteriocin which is active against a broad range of Gram-positive bacteria such as *Staphylococcus simulans*, *B. subtilis*, and *Bacillus stearothermophilus* (Linnett and Strominger, 1973; Parisot et al., 2008).

Many regulation systems mediate bacteriocin production, secretion and immunity. Bacteriocin production is usually linked to particular cellular events such as stress responses. For instance, subtilin production depends on cell density and is increased under starvation conditions (Abriouel et al., 2011). Lantibiotic production is also mediated by QS. For subtilin, it has been demonstrated that the peptide itself acts as an auto-inducer of its own production (Kleerebezem, 2004). The export of bacteriocins is generally ensured by a dedicated membrane-associated ATP-Binding Cassette (ABC) transporter. For some lantibiotics, the cleavage of the leader peptide often occurs in a proteolytic domain present in the ABC transporter as described in McAuliffe et al. (2001) and Cotter et al. (2005). The immunity of the producing strains to its own active bacteriocin(s) can be achieved by several mechanisms like the secretion of immunity proteins sequestering the peptide, the bacteriocin re-export through an ABC transporter system or the alteration of the targeted peptidoglycans bonds (*e.g.*, modification of the cell wall or cytoplasmic membrane charge) (Cotter et al., 2005; Dubois et al., 2009).

### *B. subtilis* Group AMP Enzymes

Among the *B. subtilis* group, two major types of enzymes exhibit antagonistic activities (Supplementary Table S1): the lytic enzymes and those involved in quorum quenching (QQ). Several strains from the *B. subtilis* group have indeed been identified as capable to produce lytic enzymes with biocontrol potential (Herrera-Estrella and Chet, 1999; Kumar et al., 2012; Shafi et al., 2017). They include cellulases, glucanases, proteases and chitinases and are generally referred to as cell wall degrading enzymes (CWDE) (Ariffin et al., 2006; Alamri, 2015; Caulier et al., 2018). They are particularly active against fungi since chitin and glucan are the major constituents of their cell wall where various glycoproteins are embedded (Bowman and Free, 2006; Geraldine et al., 2013; Gomaa, 2012).

**Figure 4:**
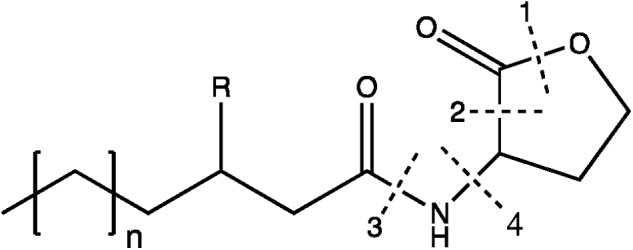
AHLs structure and its corresponding enzymatic degradations by QQ. The broken lines show the cleavages sites of four enzymes: (1) lactonase; (2) decarboxylase; (3) acylase; (4) deaminase. Adapted from Czajkowski and Jafra (2009).

Quorum quenching is able to silence or block QS which is generally defined as the cell-to-cell communication mechanism through the production of signal molecules (Czajkowski and Jafra, 2009). *N*-acyl-homoserine lactones (AHLs), composed of a fatty acid side chain and a homoserine lactone (Figure 4) are the most characterized signal autoinducers in Gram-negative bacteria. When a bacterial population proliferates, concentration of AHLs increases so that all the cells coordinate their metabolic activities (*e.g.*, biofilm formation, sporulation, virulence factors or antibiotic production) (Dong et al., 2004). As the QS system brings ecological advantages to a coordinate population, QQ is able to counteract QS. Four types of enzymes (*i.e.*, lactonase, decarboxylase, acylase, and deaminase) are able to inactivate AHLs, as illustrated in Figure 4 (Czajkowski and Jafra, 2009). *B. subtilis* AHL-lactonases have for instance attracted interest for biocontrol since they affect the growth of deleterious microbial pest such as *Pectobacterium carotovorum* subsp. *carotovorum* causing potato soft rot (González and Keshavan, 2006).

## Polyketides

**Figure 5:**
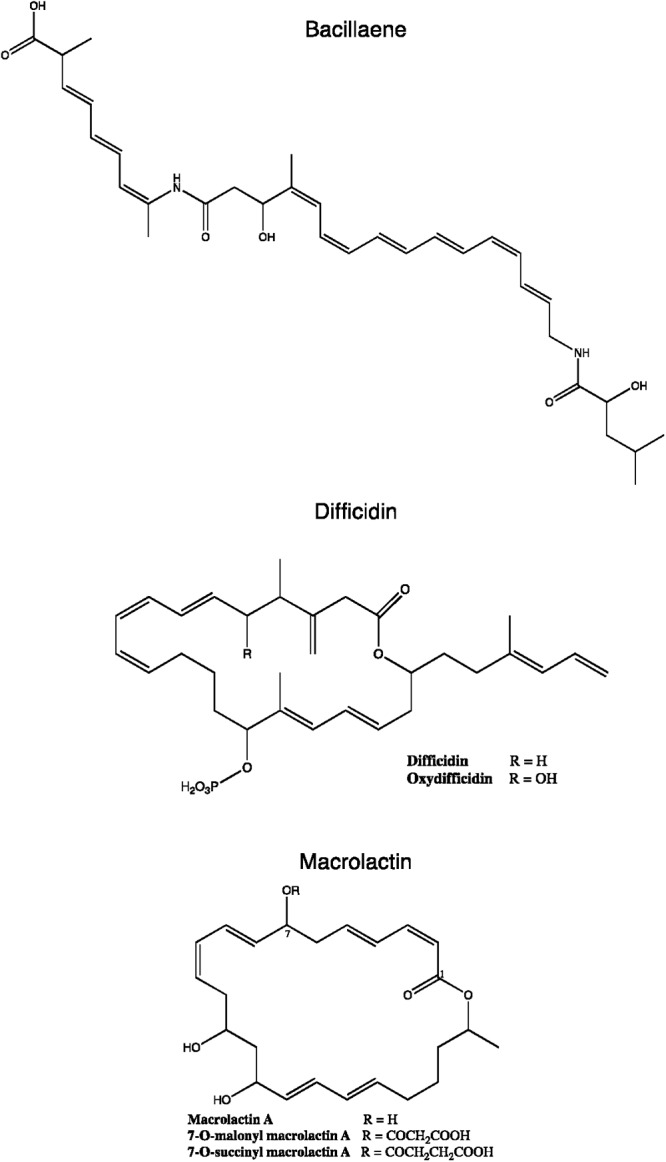
Chemical structures of some *B. subtilis* group polyketides. Variants from macrolactin and difficidin are presented.

**Figure 6:**
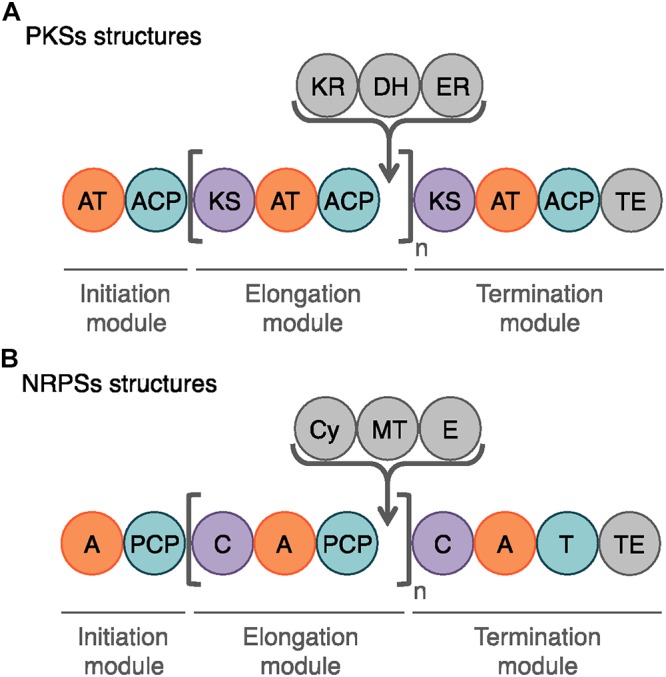
Schematic representation of the modules and domains mediating PKS and NRP biosynthesis. **(A)** The domains involved in the PK synthesis are the acyltransferase (AT), the acyl carrier protein (ACP), the ketosynthase (KS) and the chain-terminating thiosterase (TE) domains. In gray, the auxiliary domains can mediate ketoreduction (KR), dehydration (DH), and enoylacyl reduction (ER) at each elongation step (n). **(B)** The core domains for NRP biosynthesis are the adenylation (A), the peptidyl carrier domain (PCP), the condensation (C), and the final thioesterase (TE) domains. The auxiliary domains consist in cyclization (Cy), *N*-methylation (MT), and epimerization (E) domains.

**Figure 7:**
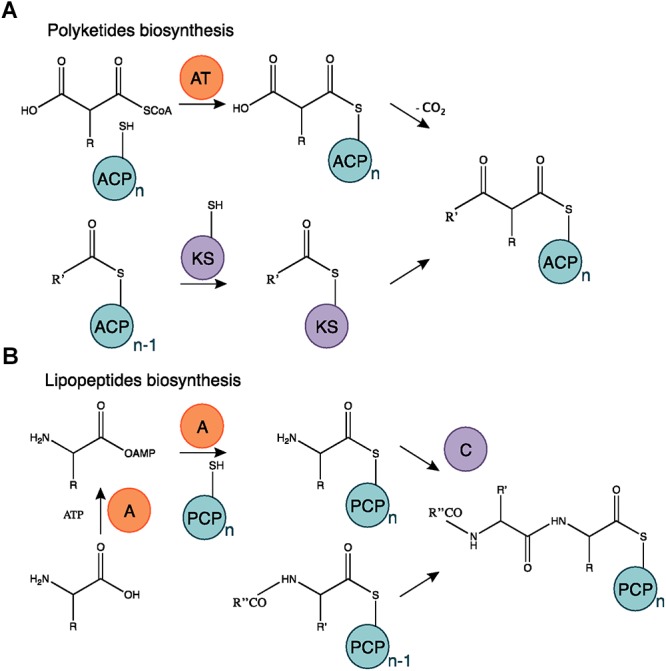
Polyketides and lipopeptides biosynthesis mechanism. **(A)** The AT domain catalyzes the binding of the monomer substrate and the ACP domain. The KS domain is acetylated on the acyl residue of a polyketide starter or in elongation and catalyzes the transfer of the substrate subunit carried by the ACP. **(B)** The A domain activates an AA chain extension subunit and its transfer to the PCP carrier domain. The C domain catalyzes the bond mediating the chain elongation. Adapted from Cane and Walsh (1999) and Challis and Naismith (2004).

Among the bioactive compounds produced by microorganisms, PKs are well known from the human health sector for their broad spectrum of activity encompassing antibacterial, immunosuppressive, antitumor and many more antagonistic abilities. Typical PKSs structures from the *B. subtilis* group are presented in Figure 5. They are synthetized from acyl CoA precursors such as malonate and methyl malonate. Their biosynthesis depends on multifunctional polyketide synthases (PKSs). Their structure was first extrapolated from fatty acid synthases (FASs) that share similarities in terms of chain extension mechanisms, precursors and overall architecture design (Smith and Tsai, 2007). As shown in Figure 6A, PKS are composed of a succession of elongation modules, flanked by initiation and termination modules. The reactive mechanism of these three PKS domains is illustrated in Figure 7A and is well summarized in Hertweck (2009). The initiation module is composed of two domains: an acyltransferase (AT) domain that recruits and catalyzes the binding of a monomer substrate to an acyl carrier protein (ACP) domain. The ACP then acts as an arm with a second catalytic domain located on the next elongation module. This domain, a β-ketoacyl synthase (KS), catalyzes the chain-elongation reaction that occurs through a decarboxylative Claisen thioester condensation (Cane and Walsh, 1999; Hertweck, 2009). In addition to the three core domains, auxiliary domains can also be present on elongation modules (gray domains in Figure 6A). These auxiliary domains mediate ketoreduction (KR), dehydration (DH), or enoylacyl reduction (ER) occurring before the chain-elongation reaction. These modifications considerably enrich the structural complexity and diversity of mature PKs (Hertweck, 2009). Finally, a termination module harboring an additional thiosterase (TE) domain catalyzes the macrolactonization and the release of the mature PK (Cane and Walsh, 1999).

Polyketide synthases have been classified in three canonical types based on the structural organization of their functional domains. Type I PKSs involve large multifunctional enzymes housing several domains linearly arranged and covalently bonded. Type II PKSs are multienzyme complexes composed of separate monofunctional enzymes combined during the PK synthesis. Type III PKSs are chalcone synthase-like PKSs that operate the acid CoA thioesters directly without any ACP domain (Chen and Du, 2016). Beside these structural differences, PKSs are classified as iterative or non-iterative depending on how many KS domains are used in the biosynthetic process. Within prokaryotes, the non-iterative type I PKSs is the most represented. They produce PK compounds that harbor a one-to-one correspondence with the PKS modular architecture. This conservation of collinearity is used for PKS discovery via genome mining (Challis, 2008).

Due to the diversity of PKSs, many exceptions and transition states between the three main types are observed. In some cases, mixed PKs pathways combine different types of PKSs or can even be associated with FASs or NRP synthetases (NRPSs) to form PK-peptide hybrid metabolites such as bacillaene, compactin, fusarin C or salinosporamide A (Moldenhauer et al., 2007; Hertweck, 2009; Fisch, 2013).

To date, seven PKs families have been recognized based on their carbon skeletons and typical structures, as summarized in Table 2 (Eustáquio et al., 2009). However, to our knowledge, only three antimicrobial PKs and their variants are produced within the *B. subtilis* group: bacillaene, difficidin, and macrolactin. These compounds exhibit antibacterial activities through selective inhibition of protein synthesis (Table 3). Bacillaene is a polyene PK resulting from a hybrid synthesis by a type I PKS and a NRPS *bae* operon (*baeJ, baeL, baeM, baeN* and *baeR*) (Chen et al., 2006; Moldenhauer et al., 2007). Its exhibits antimicrobial activity against various bacteria (*e.g.*, *Myxococcus xanthus* or *Staphylococcus aureus*) and fungi (*e.g.*, *Trichoderma* spp. or *Fusarium* spp.) (Patel et al., 1995; Um et al., 2013; Müller et al., 2014). Difficidin, and its oxidized form oxydifficidin, are polyenes synthesized by a type I PKS encoded in the *dif* operon. They both inhibit bacterial pathogens such as *Clostridium perfringens, Erwinia amylovora, Escherichia coli* or *Xanthomonas oryzae* (Zimmerman et al., 1987; Chen et al., 2009; Aleti et al., 2015; Wu et al., 2015b). Finally, macrolactins and their 7-*O*-succinyl- or 7-*O*-malonyl-derivatives are synthetized via a type I PKS. They show antibacterial and antifungal activities against *Burkholderia cepacia*, *Ralstonia solanacearum*, *S. aureus* or *Fusarium oxysporum* (Romero-Tabarez et al., 2006; Yoo et al., 2006; Yuan et al., 2012a). Some macrolactins, such as the macrolactin A, apparently also displays antiviral properties (*e.g.*, against Herpes simplex viruses) (Gustafson et al., 1989).

**Table 2:** Major classes of polyketides.

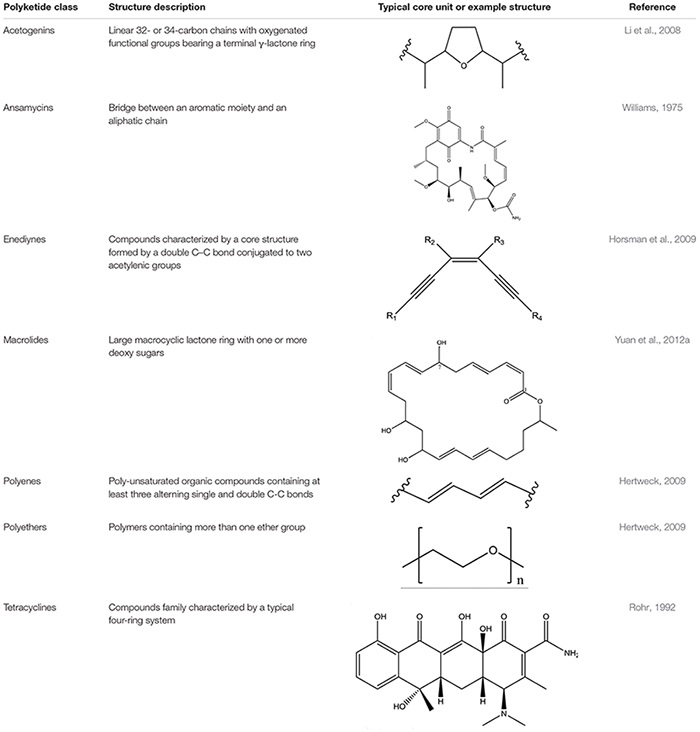

**Table 3:** PKS and hybrids NRPS/PKS produced by strains of the *B. subtilis* group.

PKS or hybrids class^∗^	Compound	Antimicrobial activity^∗∗^		References
		Antibacterial activity	Antifungal activity	
Macrolides	7-*O*-malonyl-macrolactin A	*B. cepacia*^c^*, Enterococci faecalis*^c^*, R. solanacearum*^c^*, S. aureus*^c^	*F. oxysporum* f. sp. *cubense*^c^	Romero-Tabarez et al., 2006; Yuan et al., 2012a
Macrolides	7-*O*-succinyl-macrolactin F	*B. subtilis*^c^*, S. aureus*^c^	–	Jaruchoktaweechai et al., 2000; Nagao et al., 2001
Macrolides	7-*O*-succinyl-macrolactin A	*B. subtilis*^c^*, R. solanacearum*^c^*, S. aureus*^c^	*F. oxysporum* f. sp. *cubense*^c^	Jaruchoktaweechai et al., 2000; Yuan et al., 2012a
Macrolides	Macrolactin A	*R. solanacearum*^c^	*F. oxysporum* f. sp. *cubense*^c^	Yuan et al., 2012a
Macrolides	Macrolactin D	*S. aureus*^c^	*A. solani*^c^*, Pyricularia oryzae*^c^	Xue et al., 2008
Macrolides	Macrolactin F, G, H, I, J, K, L, M	*B. subtilis*^c^*, S. aureus*^c^	–	Jaruchoktaweechai et al., 2000; Nagao et al., 2001
Macrolides	Macrolactin N	*E. coli*^c^*, S. aureus*^c^	–	Yoo et al., 2006
Macrolides	Macrolactin Q	*B. subtilis*^c^*, E. coli*^c^*, P. aeruginosa*^c^*, S. aureus*^c^	–	Mojid Mondol et al., 2011
Macrolides	Macrolactin S	*B. subtilis*^c^*, E. coli*^c^*, S. aureus*^c^	*P. oryzae*^c^	Lu et al., 2008
Macrolides	Macrolactin T	*S. aureus*^c^	*A. solani*^c^*, P. oryzae*^c^	Xue et al., 2008
Macrolides	Macrolactin W	*B. subtilis*^c^*, E. coli*^c^*, P. aeruginosa*^c^*, S. aureus*^c^	–	Mojid Mondol et al., 2011
Polyenes	Bacillaene A	*B. thuringiensis*^c^*, E. coli*^c^*, Klebsiella pneumoniae*^c^, *M. xanthus*^c^*, P. vulgaris*^c^*, Serratia marcescens*^c^*, S. aureus*^c^	*Coriolopsis* spp.^c^, *Fusarium* sp.^c^, *Pseudoxylaria* sp.^c^, *Trichoderma* sp.^c^, *Umbelopsis* sp.^c^	Patel et al., 1995; Um et al., 2013; Müller et al., 2014
Polyenes	Difficidin	*Actinomyces naeslundii*^c^*, Bacteroides distasonis*^c^*, C. perfringens*^c^*, E. amylovora*^c^*, E. coli*^c^*, Eubacterium limosum*^c^*, K. pneumoniae*^c^*, P. vulgaris*^c^*, P. aeruginosa*^c^*, S. marcescens*^c^*, S. aureus*^c^*, Streptococcus faecalis*^c^*, X. oryzae*^c^	–	Zimmerman et al., 1987; Chen et al., 2009; Wu et al., 2015b
Polyenes	Oxydifficidin	*A. naeslundii*^c^*, B. distasonis*^c^*, C. perfringens*^c^*, E. coli*^c^*, E. limosum*^c^*, K. pneumoniae*^c^*, P. vulgaris*^c^*, P. aeruginosa*^c^*, S. marcescens*^c^*, S. aureus*^c^*, S. faecalis*^c^	–	Zimmerman et al., 1987
Hybrids PKs/NRPs	Kanosamine	–	*C. albicans*^p^*, Saccharomyces cerevisiae*^p^	Janiak and Milewski, 2001; van Straaten et al., 2013

^c^*Activity of isolated compound confirmed by compound purification or mutant deletion, ^*p*^ putative activity of the compound contained in a broth mixture. ^∗^ Two PKs classes are reported in this review (macrolides and polyenes) as well as the hybrids between PKs and NRPs. ^∗∗^ -, no activity known*.

## Non-Ribosomal Peptides

Non-ribosomal peptides form a versatile family of secondary metabolites with growing interest in many industrial fields as antibiotics, siderophores, surfactants, pigments, immunosuppressors or antitumor molecules (Wang et al., 2014). NRPs show a broad structural diversity, from linear to cyclic or branched structures (Kopp and Marahiel, 2007). As illustration, the Norine database counts almost 1.200 NRP molecules, including their structure, synthesis and evolution^2^ (last update in January 2019) (Caboche et al., 2008).

**Figure 8:**
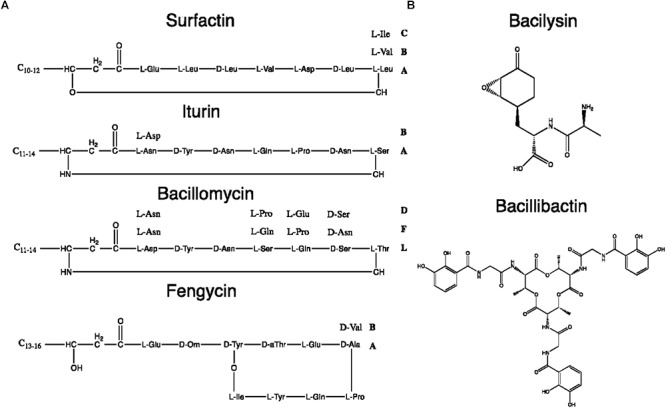
Chemical structures of some *B. subtilis* group NRPs. **(A)** Lipopeptides. **(B)** Miscellaneous NRPs.

Two categories of NRPs can be distinguished whether they are synthetized through a multi-enzyme thio-template mechanism or not (Sumi et al., 2015). The first ones usually result in structures with two to *ca.* 50 residues and other moieties such as fatty acid chains [*i.e.*, lipopeptides (LPs) and siderophores] whereas the second ones are generally smaller. Figure 8 shows the chemical structures of typical NRPS from the *B. subtilis* group.

### Thiotemplate NRPs – Lipopeptides

Lipopeptides are usually synthetized through a NRPS sequential addition of AA residues, either in an iterative or non-iterative way. Similarly to PKSs, NRPSs have a modular organization implementing the initiation, elongation, and termination modules (Figure 6B). Each module is subdivided in core domains whose catalytic and carrier domains slightly differ from PKSs, as shown in Figure 7B. The biosynthesis which was previously summarized in Ongena and Jacques (2008) and Raaijmakers et al. (2010) starts with an adenylation domain (A domain) that recruits and phosphorylates an AA monomer into an aminoacyl adenylate intermediate. The intermediate is then linked to the corresponding peptidyl carrier protein or thiolation domain (PCP or T domain) through a thioester bond. The PCP acts as a bridge and ensures the link with the condensation domain (C domain) that forms the C–N bond between the recruited aminoacyl and the peptide acyl chain in formation. The termination module contains a thioesterase domain (TE) that catalyzes the release of the final peptide acyl chain (Ongena and Jacques, 2008; Raaijmakers et al., 2010). The elongation modules can be supplemented with accessory domains such as cyclization domain (Cy), epimerization domain (E) and methylation domain (M). Those domains are able to modify the growing peptide chain which leads to diverse mature compounds structure (Cane and Walsh, 1999; Challis and Naismith, 2004).

Since the LP biosynthetic pathways are highly flexible, the range of produced LPs is extremely heterogeneous. Among LPs produced by *Bacillus* spp., four main families have been distinguished: kurstakins, surfactins, iturins, and fengycins (Jacques, 2011). Each family shares the same structural features based on the nature and organization of the peptide moiety or fatty acid tail, as summarized in Table 4. Strains from the *B. subtilis* group produce surfactins, iturins and fengycins whereas kurstakins are produced by *B. thuringiensis* strains (Béchet et al., 2012). Among the three LP families produced by *B. subtilis*, at least eight fengycins, 13 surfactins and 14 iturins variants have been described so far, as detailed in Supplementary Table S2.

**Table 4:** Classification of the *Bacillus* spp. lipopeptides.

Family^∗^	Surfactin	Iturin	Fengycin	Kurstakins
Peptide length	Heptapeptide	Heptapeptide	Decapeptide	Heptapeptide
Chiral sequence	LLDLLDL	LDDLLDL	LDDDLDLLLL	Not described
FA type	β-hydroxy FA	β-amino FA	β-hydroxy FA	β-hydroxy FA or not
FA length	13–15 carbons	14–17 carbons	16–19 carbons	11–14 carbons
Structure	Cyclic lactone	Cyclic peptide	Cyclic lactone	Cyclic lactone

^∗^*FA refers to fatty acid*.

For each LP family, the compounds production is mainly regulated by environmental factors such as carbon sources, oxygen availability, pH and temperatures (Yakimov et al., 1995; Kim et al., 1997; Cosby et al., 1998). Warm temperature (≥37°C) and anaerobic conditions increase the production of surfactins while lower temperatures (25–37°C) and aerated bioreactors favor fengycins and iturins family metabolites (Jacques, 2011). The production of surfactins by *B. subtilis* is also QS-dependent and involves ComX and PhrC. These pheromones trigger complex cascades regulating cell density-dependent processes such as sporulation and competence (Hamoen et al., 2003; Ongena et al., 2005).

Iturins and fengycins are mainly known for their strong antifungal activity against several plant and human pathogenic fungi (Supplementary Table S2). In addition, iturin-like mycosubtilin, bacillomycin R, subtulene A and eumycin show antibacterial properties (Besson et al., 1976; Leclere et al., 2005; Thasana et al., 2010). Contrary to iturins and fengycins, surfactins mainly display antiviral and antibacterial activities (Ongena and Jacques, 2008). Their antiviral activity essentially targets enveloped viruses (*e.g.*, herpes simplex or porcine epidemic diarrhea viruses). They also inhibit pathogenic bacteria such as *Legionella pneumophila*, *Listeria monocytogenes*, *R. solanacearum* or *X. oryzae* (Naruse et al., 1990; Yakimov et al., 1995; Sabaté and Audisio, 2013; Loiseau et al., 2015; Luo et al., 2015). However, some surfactins are able to control important fungal plant and human pathogens such as *Botrytis cinerea, Candida albicans, F. oxysporum* or *Rhizoctonia solani* (Jenny et al., 1991; Lee et al., 2007; Qi et al., 2010; Dimkić et al., 2013; Romano et al., 2013).

The mere composition of LPs, where a peptide moiety is bound to a lipid tail, gives them an amphiphilic property. This nature makes them excellent surfactants and plays a significant role in their biological functions and antimicrobial properties. Indeed, LPs are able to destabilize the plasma membrane via a pore forming activity leading to the cell death of the target microbes. Their antiviral activity is the result of a similar disintegration of the bi-lipid envelope of virions explaining the weak LPs activity against plant viruses among which very few are enveloped (Ongena and Jacques, 2008).

*Bacillus* spp. LPs have many other biological and ecological functions as fully documented by Raaijmakers et al. (2010). They are also known to impact other metabolic mechanisms such as biofilm formation, motility, virulence, plant root colonization, and plant defenses. Moreover, it has been suggested that their participation to the degradation of hydrophobic substrates could be used for polluted soils bioremediation (Mulligan et al., 2001). Although some lipopetides have already been exploited as food biopreservatives or crop protection products, the industrial interest for LPs in specific applications is unsurprisingly continuously growing.

### Thiotemplate NRPs – Siderophore

Itoic acid is a mono-peptide composed of a 2,3-dihydroxybenzoate (DHB) molecule bound to a glycine. It is used as a precursor by trimodular NRPS machinery to produce bacillibactin which is obtained after a condensation of three units of DHB-glycine-threonine (May et al., 2001). The synthesis of the final hexapeptide is catalyzed by a terminal thioesterase domain leading to the production of a methylated trilactone ring link to three catecholates moieties. It is this cyclic structure that enables the sequestration of the metal atom (Dertz et al., 2006). Itoic acid and bacillibactin are both catecholic siderophores that chelates iron reducing its bioavailability. This is limited access to iron that allows *B. subtilis* to antagonize the growth of other surrounding microbes such as, for instance, *F. oxysporum* f. sp. *capsici* (Yu et al., 2011).

### Non-thiotemplate NRPs

Bacteria from the *B. subtilis* group are also able to synthesize other antimicrobial NRPs through non-thiotemplate mechanism. Rhizocticins are di- and tri-phosphono-peptides. They are constituted of a L-2-amino-5-phosphono-3-*cis*-pentenoic acid (APPA) linked to an arginine (rhizocticin A). They can be supplemented with an additional valine (rhizocticin B), isoleucine (rhizocticine C) or leucine (rhizocticine D). After their integration into the target microbes, their cleavage by host cell peptidases releases the fungitoxic L-APPA moiety that interferes with threonine metabolism in fungal cells. Interestingly, rhizocticin A has also an antagonistic activity against nematodes such as *Caenorhabditis elegans* (Kugler et al., 1990).

In addition to rhizocticin compounds, two other dipeptide NRPs are produced by *B. subtilis*: bacilysin (also known as tetaine) and its chlorinated derivative, chlorotetain. They contain L-alanine (or chlorine-L-alanine) bound to the non-proteinogenic L-anticapsin (Kenig and Abraham, 1976; Rapp et al., 1988). Despite their simple composition, these bioactive compounds display strong antibacterial activity mediated by the anticapsin moiety that inhibits the glucosamine-6-phosphate synthase. Its inhibition suppresses the biosynthesis of peptidoglycans that are the main constituents of bacterial cell wall (Steinborn et al., 2005; Mahlstedt and Walsh, 2010). For the fungi, it has been proposed that because anticapsin is able to inhibit the production of chitin and fungal membrane mannoproteins, bacilysin and chlorotetain exhibit antifungal activity against *Aspergillus fumigatus* or *C. albicans* (Milewski et al., 1986; Rapp et al., 1988).

Finally, bacitracin and mycobacillin are two cyclic polypeptides produced by *B. subtilis*. Bacitracins are dodecapeptides containing a cyclic heptapeptide linked to a thiazoline ring (Johnson et al., 1945). They are mostly active against Gram-positive bacteria where they inhibit the bacterial cell-wall biosynthesis by preventing the lipid carrier from re-entering in the reaction cycle of peptidoglycan synthesis (Siewert and Strominger, 1967). Besides this primary mode of action, bacitracin might also act through other mechanisms affecting membrane functions, hydrolytic enzymes and/or the biosynthesis of ubiquinone precursors (Konz et al., 1997). Mycobacillin is an antifungal cyclic tridecapeptide altering the membrane of fungi like *Aspergillus niger* (Majumdar and Bose, 1958). Interestingly, its biosynthesis is rather peculiar. Although it is catalyzed by a large NRPS complex, it is divided in three fractions (A, B, and C) and does not use a thio-template mechanism (Zuber et al., 1993). Each fraction of the enzymatic complex contains a single enzyme polypeptide that catalyzes the polymerization of a first pentapeptide (A), a second nonapeptide (B) and the final tridecapeptide.

## Volatiles

Besides RPs, NRPs and PKs, strains from the *B. subtilis* group are able to produce a wide diversity of volatile compounds encompassing important roles especially in soil, one of the major habitats of this group (Supplementary Figure S1). Volatiles are notably involved in the bioconversion of the food chain, in the biogeochemical cycles of essential elements, in many physiological and metabolic reactions (*e.g.*, nitrification, nitrogen mineralization, electron acceptor or donor reactions) as well as in communication signals triggering QS/QQ or defense mechanisms well reviewed in Effmert et al. (2012). Volatile compounds are generally classified into inorganic (VICs) and organic (VOCs) categories.

### Volatile Inorganic Compounds (VICs)

Volatile inorganic compounds synthesized by microorganisms are mainly by-products of primary metabolism. They are carbonated, hydrogenated, sulfur or nitrogen-containing compounds such as CO_2_, CO, H_2_, HCN, H_2_S, N_2_, NH_3_ and NO. Nitrogen-containing compounds are mostly released in aerated upper sediments layers by denitrifying bacteria. In this process, nitric oxide is enzymatically produced by the nitric-oxide reductase or the nitric-oxide synthase (Adak et al., 2002). The range of antimicrobial activities exhibited by VIC nitrogen-containing compounds from the *B. subtilis* group is wide. For instance, NO is able to induce systemic acquired resistance (SAR) in plants against bacterial pathogens such as *R. solanacearum* (Wang et al., 2005). *A contrario*, ammonia, a secondary metabolite from the catabolism of the amino acids L-aspartate, is known to be active against soil-borne Oomycetes such as *Pythium* spp. (Howell et al., 1988). Hydrogen cyanide, derived from the glycine catabolism, shows a direct antagonistic activity against aerobic microorganisms by inhibiting metal-containing enzymes such as the cytochrome c oxidase active in the respiration chain (Cherif-Silini et al., 2016).

Deeper in the soil, under low oxygen concentration, bacteria tend to produce different VICs such as H_2_ or H_2_S. Those compounds can serve as electron acceptors, AA precursors or antimicrobial metabolites. Hydrogen sulfide could be produced by *B. subtilis* from sulfate reduction or as a by-product of L-methionine and L-cysteine catabolism via a direct cleavage of L-methionine or a transamination followed by reductive demethiolations (Even et al., 2006; Schulz and Dickschat, 2007). It is known to exhibit antifungal activity against several plant pathogens such as *A. niger* or *Penicillium italicum* but also against some food-borne bacteria or human pathogens (Fu et al., 2014). Curiously, it is also known to act as a bacterial defense mechanism against antibiotics (Shatalin et al., 2011). Interestingly, ammonia increases the resistance of several Gram-negative and Gram-positive bacteria to antibiotics too (Bernier et al., 2011).

### Volatile Organic Compounds (VOCs)

Volatile organic compounds are small compounds with fewer than 20 carbon atoms and are characterized by low molecular mass (100–500 Da), high vapor pressure, low boiling point and a lipophilic moiety. These features ensure an easy evaporation and a long distance distribution which is convenient in a complex matrix like soil (Schmidt et al., 2015). Their diffusion and production by soil-borne microbes are strongly dependent on various factors such as nutrient and oxygen availability, temperature, pH, physiological state of microorganisms, soil moisture, texture and architecture (McNeal and Herbert, 2009; Insam and Seewald, 2010; Effmert et al., 2012). The majority of VOCs derives from glucose oxidation involving glycolysis and the subsequent cycles such as the tricarboxylic acid cycle (TCA) as it has been well summarized in Korpi et al. (2009) and Schmidt et al. (2015). However, their production can also result from various other pathways such as aerobic heterotrophic carbon metabolism, fermentations, AA degradation, terpenes synthesis or sulfur reduction (Peñuelas et al., 2014). Based on previous reviews presented in Schulz and Dickschat (2007); Peñuelas et al. (2014) and Audrain et al. (2015), five categories of VOCs can be distinguished: (1) fatty acids and derivatives, (2) terpenoids, (3) nitrogen-containing VOCs, (4) sulfur-containing VOCs, and (5) metalloid- or halogenated-containing VOCs. To date, about 2,000 compounds produced by almost 1,000 species of microorganisms have been listed in the mVOC 2.0 database (Lemfack et al., 2018). According to this database, almost 70% of recorded *Bacillus* VOCs are fatty acids derivatives (alcohols, ketones, alkanes, aldehydes, alkenes, and acids) followed by sulfur- and nitrogen-containing compounds. Supplementary Table S3 displays the VOCs produced within the *B. subtilis* group and their antimicrobial activity.

Since many volatile fatty acids and their derivatives result from the glucose metabolism, their precursors mostly derive from the Embden-Meyerhof (glycolysis), Entner-Doudoroff, heterolactic and homolactic fermentation pathways (Peñuelas et al., 2014). *B. subtilis* bacteria, for instance, ferment pyruvate to produce ketone compounds such as acetoin (3-hydroxy-2-butanone) or 2,3-butanedione under anaerobic conditions (Ryu et al., 2003). Other intermediates coming from fatty acid biosyntheses or their β-oxydations are also used as precursors by microbes and transformed into VOCs through a decarboxylation reaction or a reduction of their carboxyl group (Schulz and Dickschat, 2007). They provide essential hydrocarbons but also other fatty acid derivatives. An oxidative deamination of several amino acids can lead to the production of aldehyde, ketone or alcohol volatile too. For instance, the degradation of L-phenylalanine or L-tyrosine can be the first step of the aromatic volatile compounds synthesis such as benzene or its carbohydrate derivatives. Finally, benzenoid volatiles can also be synthesized by microbes through the shikimate pathway that leads to the formation of chorismate, a natural precursor of aromatic amino acids (Bentley and Haslam, 1990). Degradation of intermediates from the shikimate pathway or aromatic amino acids can also lead to the production of benzenoid volatiles (Dickschat et al., 2005).

This wide variety of volatile fatty acids and their derivatives make them the most important group of VOCs produce by microbes and represent up to 87% of known antimicrobial VOCs produced by *B. subtilis* bacteria (Supplementary Table S3). They can be divided in two main categories: hydrocarbons (alkanes, alkenes, alkynes) or carbohydrates (acids, alcohols, aldehydes, esters, furans, ketones, lactones, benzenoids). Among them, benzenoids is the most represented sub-category followed by alkanes, aldehydes, ketones, acids, and alcohols. Even though benzenoids could be considered as an individual category, they can also be seen as fatty acids derivatives because a large majority of antimicrobial benzenoid volatile produced by *B. subtilis* harbor a benzene core linked to a fatty acid derivatives.

There is an important diversity of benzenoids, sometimes linked with carbohydrate chains containing nitrogen, sulfur or both. Most of these antimicrobial volatile exert fungicidal activities but some have been characterized for their antibacterial or nematicidal abilities, too. Their mode of action is rarely fully characterized. For instance, morphological abnormalities on fungal and bacterial cells have been documented after an exposition to *B. subtilis* VOCs (Tahir et al., 2017). Volatile such as 1,3-butadiene or 2,3-butanediol are also known to induce modifications in the expression of genes linked to the pathogenicity of *R. solanacearum* and *Pectobacterium carotovorum* (Marquez-Villavicencio et al., 2011; Tahir et al., 2017). In addition to direct antimicrobial activities, fatty acids volatile have also several other biological functions. For instance, acetoin and 2-butanone have the ability to stimulate plant defenses or to induce plant stress tolerance which then promote plant growth (Ryu et al., 2003; Ryu et al., 2004; Ryu, 2015). They are essentially produced by strains of *B. amyloliquefaciens*, *B. velezensis* or *B. subtilis* (Audrain et al., 2015).

Terpenes and their derivatives (also known as terpenoids or isoprenoids) are among the most abundant secondary metabolites found in living systems (Fisher et al., 2001; Gershenzon and Dudareva, 2007). They originate from two main precursors: isopentenyl pyrophosphate (IPP) and its allylic isomer the dimethylallyl pyrophosphate (DMAPP) (Schulz and Dickschat, 2007). IPP and DMAPP are also the end-products of the deoxy-xylulose phosphate pathway (DOXP) starting with pyruvate and glyceraldehyde-3-phosphate originating from the glucose metabolism (Fisher et al., 2001). Terpenoids can be synthesized from isoprene molecules too. Julsing et al. (2007) showed that, in *B. subtilis*, isoprene is not formed by the MVA or DOXP pathways but, as in plant systems, might be a product of the methylerythritol phosphate (MEP) pathway (Guan et al., 2015).

Isoprenoid compounds are produced by all living organisms for essential physiological functions such as electron transport, membrane fluidity, light harvesting, photoprotection, anchoring of molecules to specific membranes and signaling (Fisher et al., 2001). The signaling ability is particularly important and is associated with several antagonistic, mutualistic or multi-trophic interactions (Shrivastava et al., 2015). More than 25,000 terpenic compounds have been listed and, for the vast majority, their biological functions and roles remain unknown (Buckingham, 1997). Volatile terpenes are generally recognized for their ability to inhibit bacteria (Scortichini and Rossi, 1991), fungi (Hammer et al., 2003; Dambolena et al., 2008), nematodes (Gu et al., 2007) or insects (Lee et al., 2003; Justicia et al., 2005). They can be classified in three categories: isoprene, monoterpenes (C_10_) and sesquiterpenes (C_15_) (Schmidt et al., 2015).

The mode of action of these compounds might be linked to their lipophilic nature allowing them to destabilize the cell membrane integrity (Cox et al., 2000; Inoue et al., 2004). To our knowledge, only two terpenes produced by *B. subtilis* show antimicrobial abilities: isoprene and monoterpene α-terpineol exhibit antagonistic activities against cyanobacteria and nematodes (Wright and Thompson, 1985; Gu et al., 2007).

Little is known about the biosynthetic pathways of nitrogen-containing VOCs. Nevertheless, it is accepted that two main routes can be used: a non-enzymatic amination of acyloins, that can lead to the formation of pyrazines (Schulz and Dickschat, 2007) or derived from α-aminoketone intermediates resulting from AA catabolism (Owens et al., 1997; Zhu et al., 2010).

Nitrogen-containing VOCs can be distinguished based on their cyclization rate. Within non-cyclic compounds, three groups are identified (amides, amines and imines) while there are five categories of cyclic compounds (azoles, pyrazines, pyridines, pyridazines, and pyrimidines). Pyrazines are strongly represented among microbial volatile and are separated in two classes: lower-alkylated and higher-alkylated pyrazines (Schulz and Dickschat, 2007). These compounds are characterized by a strong odor and several *B. subtilis* coming from the rhizosphere or from food fermentations have already been recognized as pyrazines producers (Sugawara et al., 1985; Kosuge and Kamiya, 1962; Larroche et al., 1999; Leejeerajumnean et al., 2001). Pyrazines from *B. subtilis* strains are known to exhibit antifungal and nematicidal activities (Gu et al., 2007; Chen et al., 2008; Chaves-López et al., 2015; Haidar et al., 2016). For instance, tetramethylpyrazine inhibits the growth of *Moniliophthora perniciosa* and *F. oxysporum* f. sp. *lactucae*. Additionally, it acts on sporulation and elongation of the germ-tube of *B. cinerea* (Chen et al., 2008; Chaves-López et al., 2015). It is interesting to note that *B. subtilis* pyrazines can also exhibit antibacterial activities such as pulcherriminic acid which inhibits the growth of *S. aureus*, *E. coli* and *Proteus vulgaris* (Coutts et al., 1965). Beside pyrazines, strains from the *B. subtilis* group are able to produce other nitrogen VOCs such as 1H-imidazole,1-ethyl showing antifungal activities against numerous soil-borne phytopathogens (Lupetti et al., 2002; Liu et al., 2008; Snelders et al., 2009; Schmidt et al., 2015).

Microbial VOCs containing sulfur (VSCs) derive from two main pathways originated from inorganic or organic sources (Schulz and Dickschat, 2007): inorganic sulfate reduction in methylated inorganic sulfides compounds or, for some microbial VSCs, originate from catabolism of AA such as L-methionine or more rarely, L-cysteine (Schulz and Dickschat, 2007). Some VSCs are produced as secondary volatiles via the production of hydrogen sulfide or methanethiol. Indeed, these two compounds are important precursors for subsequent VSCs synthesis (Schulz and Dickschat, 2007; Sourabié et al., 2012). Within the *B. subtilis* group, multiple VSCs such as dimethyl disulfide (DMDS), dimethyl trisulfide (DMTS), *S*-methyl thioacetate or *S*-methyl butanethioate have been characterized for their antifungal and nematicidal activities (Coosemans, 2005; Gerik, 2005; Gu et al., 2007; Kai et al., 2009; Wang et al., 2009; de Vrieze et al., 2015; Schmidt et al., 2015; Velivelli et al., 2015; Gotor-Vila et al., 2017). A putative antibacterial effect of DMDS is not to exclude. Indeed, DMDS is known to affect the bacterial cell-to-cell communications through a decrease in the amount of *N*-acyl homoserine lactone (AHL) mediating QS (Chernin et al., 2011).

Other volatile organic compounds such as halogenated, metalloids, tellurium or selenium compounds have also been described. However, at the time of writing, no *B. subtilis* strains have been proved to produce these type of VOCs (Schulz and Dickschat, 2007), although related bacteria, like *Bacillus arsenicoselenatis*, have been shown to generate them (Switzer Blum et al., 1998).

## Conclusion and Perspectives

The *B. subtilis* group offers a plethora of antagonistic compounds displaying a broad range of biological functions. This huge versatility increases the industrial and environmental interest of *B. subtilis* strains, especially when considering their range of action against foodborne or phytopathogenic flora as well as their history of safe use in food. The present review on known AMCs from the *B. subtilis* group proposes a consistent classification frame based on their biosynthetic pathways (*i.e.*, RPs, PKs, NRPs, volatiles) and chemical nature.

The present classification suggests to establish systematic approaches for novel molecules discoveries and characterizations (biosynthesis, chemical nature and activity). Indeed, most current publications report antimicrobial activity of partially purified fractions which can involve mixtures of bioactive compounds. To assess the activity of an unique compound, implementations of genetic confirmation such as knockout strategy are needed. Besides, very few studies have focused on the putative synergistic effects within these bio-active mixtures. Also, the concentration of purified or semi-purified compound(s) often remains uncharacterized or biologically irrelevant. Finally, there is no doubt that novel AMCs originating from *B. subtilis* bacteria remain to be identified, characterized and properly classified.

## Author Contributions

SC, CN, and FL conducted the bibliographic search. SC and CN wrote the manuscript. AG, CB, and JM edited and reviewed the manuscript. All authors have read and approved the final version.

## Conflict of Interest Statement

The authors declare that the research was conducted in the absence of any commercial or financial relationships that could be construed as a potential conflict of interest.

## Footnotes

1 http://www.bacterio.net/bacillus.html

2 http://bioinfo.lifl.fr/norine
