# Supplementary material for: Overview of the Antimicrobial Compounds Produced by Members of the Bacillus subtilis Group
Source: Front Microbiol. 2019 Feb 26;10:302. doi: 10.3389/fmicb.2019.00302 (PMC6401651; doi:10.3389/fmicb.2019.00302)
Supplement: Supplementary file 1 [file Table_1.docx]

**Supplementary tables**

**Table S1:** Ribosomal peptides produced by strains of the *B. subtilis* group.

| **RPs class*** | **RPs subclass**** | **Compound** | **Antimicrobial activity***** | | **References** |
| --- | --- | --- | --- | --- | --- |
|  |  |  | **Antibacterial activity** | **Antifungal activity** |  |
| Bacteriocin | Subclass I.1 | Entianin | *Enterococcus faecalis* ^c^*, Micrococcus luteus* ^c^*, Staphylococcus aureus* ^c^ | *-* | Fuchs *et al.*, 2011 |
| Bacteriocin | Subclass I.1 | Ericin A | Similar to Ericin S with minor activity | *-* | Stein *et al.*, 2002 |
| Bacteriocin | Subclass I.1 | Ericin S | *B. amyloliquefaciens* ^c^*, Bacillus brevis* ^c^*, Bacillus cereus* ^c^*, Bacillus firmus* ^c^*, Bacillus polymyxa* ^c^*, Bacillus sphaericus* ^c^*, Bacillus subtilis* ^c^*, Clavibacter michiganensis* ^c^*, Lactococcus lactis* ^c^ | *-* | Stein *et al.*, 2002, Agrios, 1988 |
| Bacteriocin | Subclass I.1 | Subtilin | *B. amyloliquefaciens* ^c^*, B. brevis* ^c^*, B. cereus* ^c^*, B. firmus* ^c^*, B. polymyxa* ^c^*, B. sphaericus* ^c^*, B. subtilis* ^c^*, C. michiganensis* ^c^*, L. lactis* ^c^ | *-* | Heinzmann *et al.*, 2006, Stein *et al.*, 2002 |
| Bacteriocin | Subclass I.2 | Mersacidin | *S. aureus* ^c^*, Staphylococcus simulans* ^c^ | *-* | Brötz *et al.*, 1995 |
| Bacteriocin | Subclass I.2 | Sublancin 168 | *B. cereus* ^c^*, Bacillus megaterium* ^c^*, B. subtilis* ^c^*, S. aureus* ^c^*, Streptococcus pyogenes* ^c^ | *-* | Paik *et al.*, 1998 |
| Bacteriocin | Subclass I.3 | Lichenicidin | *B. cereus*^p^*, Bacillus halodurans*^p^*, L. lactis*^p^*, Listeria innocua*^p^*, L. monocytogenes*^p^*, S. aureus*^p^*, Streptococcus mutans*^p^*, Streptococcus pneumoniae*^p^ | *-* | Begley *et al.*, 2009 |
| Bacteriocin | Subclass I.4 | Subtilosin A | *Enterobacter aerogenes* ^c^*, E. faecalis* ^c^*, E. coli* ^c^*, Klebsiella pneumoniae* ^c^*, Kocuria rhizophila* ^c^*, L. monocytogenes* ^c^*, Porphyromonas gingivalis*^c^*, Proteus mirabilis* ^c^*, Pseudomonas aeruginosa* ^c^*, Shigella sonnei* ^c^*, S. enterica* ^c^*, S. aureus* ^c^*, Streptococcus gordonii* ^c^*, S. pyogenes* ^c^ | *-* | Shelburne *et al.*, 2007 |
| Bacteriocin | Subclass II.3 | Lichenin | *Butyrivibrio fibrisolvens*^p^*, Eubacterium ruminantium*^p^*, Lactobacillus casei*^p^*, Ruminococcus albus*^p^*, Ruminococcus flavefaciens*^p^*, Streptococcus bovis*^p^ | *-* | Pattnaik *et al.*, 2001 |
| Bacteriocin | Class III | Bac 14B | *Agrobacterium larrymoorei* ^c^*, Agrobacterium rhizogenes* ^c^*, Agrobacterium rubi* ^c^*, Agrobacterium tumefaciens* ^c^*, Agrobacterium vitis* ^c^*, B. cereus* ^c^*, B. licheniformis* ^c^*, B. subtilis* ^c^*, Pectobacterium carotovora subsp. carotovora* ^c^*, E. coli* ^c^*, M. luteus* ^c^*, P. aeruginosa* ^c^*, Pseudomonas savastanoi* pv. *savastanoi* ^c^*, Pseudomonas syringae* pv*. syringae* ^c^*, Salmonella typhimurium* ^c^*, S. aureus* ^c^ | *Alternaria solani* ^c^ | Hammami *et al.*, 2012 |
| Bacteriocin | Class III | Baciamin | *-* | *B. cinerea* ^c^*, F. oxysporum* ^c^*, Helminthosporium turcicum* ^c^*, Helminthosporium maydis* ^c^*, Mycosphaerella arachidicola* ^c^*, Pythium aphanidermatum* ^c^*, R. solani* ^c^*, Valsa mali* ^c^ | Wong *et al.*, 2008 |
| Bacteriocin | Subclass I.4 | Amylocyclicin | *B. brevis* ^c^*, B. cereus* ^c^*, B. licheniformis* ^c^*, B. megaterium* ^c^*, B. pumilus* ^c^*, B. sphaericus* ^c^*, B. subtilis* ^c^*, C. michiganensis* ^c^*, M. luteus* ^c^*, P. granivorans* ^c^*, P. polymyxa* ^c^ |  | Scholz *et al*., 2014 |
| Bacteriocin | Subclass 1.4 (Heterocyclo-anthracin family) | Sonorensin | *B. subtilis* ^c^*, E. coli* ^c^*, L. monocytogenes* ^c^*, P. aeruginosa* ^c^*, S. aureus* ^c^, *Vibrio vulnificus* ^c^ |  | Chopra *et al*., 2014, Chopra *et al.,* 2015 |
| Bacteriocin | ND | Plantazolicin | *B. brevis*^c^*, B. cereus*^c^*, B. licheniformis*^c^*, B. megaterium*^c^*, B. pumilus*^c^*, B. sphaericus*^c^*, B. subtilis*^c^*, M. luteus*^c^*, P. granivorans*^c^ |  | Scholz *et al*., 2011 |
| Bacteriocin | Class III | CAMT2 | *E. coli*^p^*, L. monocytogenes*^p^*, S. aureus*^p^*, Vibrio parahaemolyticus*^p^ |  | An *et al.* 2015 |
| Bacteriocin | Class III | Bacisubin |  | *Alternaria brassicae*^c^*, B. cinerea*^c^*, Magnaporthe grisease*^c^*, R. solani*^c^*, S. sclerotiorum*^c^ | Liu et al., 2007 |
| Bacteriocin | Subclass I.1 | Amylolysin | *B. cereus*^c^*, B.* *megaterium*^c^*, B. subtilis*^c^*, Enterococcus faecalis*^c^*, Enterococcus faecium*^c^*, E. coli*^c^*, Lactobacillus plantarum*^c^*, Listeria innocua*^c^*, Listeria ivanovii*^c^*, L. monocytogenes*^c^*, M. luteus*^c^*, P. aeruginosa*^c^*, S. aureus*^c^*, Staphylococcus epidermis*^c^*, Streptococcus agalactiae*^c^ | *Cryptococcus neoformans* ^c^*, S. cerevisiae* ^c^ | Arguelles Arias et al., 2013 |
| Enzymes | Lytic | Chitinase |  | *Aspergillus niger*^p^ | Podile and Prakash, 1996 |
| Enzymes | Quorum quenching | AHL-lactonases | *P. carotovora subsp. carotovora*^c^ |  | Pan *et al.*, 2008 |

^c^ Activity of isolated compound confirmed by compound purification or mutant deletion, ^p^ putative activity of the active compound contained in a broth mixture.

* Two RPs classes are reported in this review: the bacteriocins and the enzymes

** See Fig. 2 for the RPs subclasses, ND: Not determined

*** - = no activity known

**Table S2:** NRPs produced by strains of the *B. subtilis* group.

| **NRPs class*** | **NRPs subclass**** | **Compound** | **Antimicrobial activity***** | | | **References** |
| --- | --- | --- | --- | --- | --- | --- |
|  |  |  | **Antibacterial activity** | **Antifungal activity** | **Antiviral activity** |  |
| Lipopeptides | Fengycin | Agrastatin A | - | *Alternaria solani* ^p^*, Botrytis cinerea* ^p^*, Plasmopara viticola* ^p^ | - | Lee and Kim, 2015 |
| Lipopeptides | Fengycin | Fengycin | - | *A. solani* ^c^*, B. cinerea* ^p^*, Fusarium graminearum* ^c^*, Fusarium sambucinum* ^c^*, F. oxysporum* ^p^*, Podosphaera fusca* ^c^*, Pythium sulcatum* ^c^*, Pythium ultimum* ^p^*, R. solani* ^c^*, Rhizopus* sp*.*^p^*, Sclerotinia sclerotiorum* ^c^ | - | Cawoy *et al.*, 2014, Ongena *et al.*, 2005, Ramarathnam *et al.*, 2007 , Romero *et al.*, 2007, Guo et al., 2014, Wise *et al.*, 2014, Zhao *et al.*, 2014 |
| Lipopeptides | Fengycin | Fengycin A | - | *F. oxysporum f. sp. radicis-lycopersici* ^c^*, F. oxysporum* f. sp*. spinaciae* ^c^*, Fusarium solani* ^p^*, S. sclerotiorum*^p^ | - | Li *et al.*, 2012 , Malfanova *et al.*, 2012, Zhao *et al.*, 2012 |
| Lipopeptides | Fengycin | Fengycin B | - | *F. oxysporum*f. sp*. radicis-lycopersici* ^c^*, F. solani* ^p^ | - | Malfanova *et al.*, 2012, Li *et al.*, 2012 |
| Lipopeptides | Fengycin | Fengycin C, D, S | - | *F. solani* ^p^ | - | Li *et al.*, 2012 |
| Lipopeptides | Fengycin | Plipastatin | - | *F. oxysporum* f. sp*. cucumerinum* ^p^, *Fusarium graminearum* ^c^ | - | Gao L. *et al.*, 2017, Gong *et al.*, 2015 |
| Lipopeptides | Iturin | Bacillomycin | - | *P. fusca* ^c^ | - | Romero *et al.*, 2007 |
| Lipopeptides | Iturin | Bacillomycin D | - | *Alternaria alternata* ^c^*, A. solani* ^c^*, Aspergillus flavus* ^c^*, Botryosphaerica ribis* ^c^*, C. albicans* ^p^*, Cryphonectria parasitica* ^p^*, Colletotrichum acutatum* ^c^*, Colletotrichum gloesporioides* ^c^*, Didymella bryoniae* ^c^*, F. graminearum* ^c^*, F. oxysporum* ^c^*, H. maydis* ^c^*, Monilinia fructicola* ^c^*, Penicillium expansum* ^c^*, Phomopsis gossypii* ^c^*, Phytophthora capsici* ^p^*, Pyricularia grisea* ^c^*, R. solani* ^c^*, Sclerotium rolfsii* ^c^*, S. sclerotiorum* ^c^ | - | Moyne *et al.* 2001, Gong *et al.*, 2014, Zhao *et al.*, 2010 , Yuan *et al.*, 2012a, Tanaka *et al.*, 2014 |
| Lipopeptides | Iturin | Bacillomycin F | - | *A. niger* ^p^*, B. cinerea* ^p^*, Byssochlamys fulva* ^p^*, F. oxysporum* ^p^*, Monascus sp.* ^p^*, Mycosphaerellla pinodes* ^p^*, Neurospora crassa* ^p^*, Penicillium chrysogenum* ^p^*, Pleospora herbarum* ^p^*, Rhodotorula pilimanae* ^p^*, Rhizopus oligosporus* ^p^*, S. cerevisae* ^p^, *Sclerotinia fructigena* ^p^*, S. sclerotiorum* ^p^*, Stemphylium radicinum* ^p^*, Trichophyton mentagrophytes* ^p^ | - | Mhammedi *et al.*, 1982,  Lee *et al.*, 2008, Thimon *et al.*, 1992 |
| Lipopeptides | Iturin | Bacillomycin L | - | *S. cerevisae* ^p^*, R. solani* ^c^ | - | Thimon *et al.*, 1992, Zhang *et al.*, 2013 |
| Lipopeptides | Iturin | Bacillomycin LC | - | *Ceratocystis fagace* ^c^*, C. parasitica* ^c^*, Ophiostoma ulmi* ^c^*, S. cerevisae* ^p^, *Verticillium dahliae* ^c^ | - | Eshita *et al.*, 1995, Besson *et al.,* 1979 |
| Lipopeptides | Iturin | Bacillomycin R | *Agrobacterium tumefaciens* ^p^ | *Penicillium chrysogenum* ^p^*, Penicillium notatum* ^p^ | - | Besson *et al.*, 1976 |
| Lipopeptides | Iturin | Eumycin | - | *P. chrysogenum* ^p^*, P. notatum* ^p^ | - | Besson *et al.*, 1976 |
| Lipopeptides | Iturin | Iturin | - | *B. cinerea* ^c^*, F. oxysporum*^c^*, P. ultimum* ^p^*, R. solani* ^p^*, Rhizopus* sp. ^p^ | - | Ongena *et al.*, 2005, Cawoy *et al.*, 2014 |
| Lipopeptides | Iturin | Iturin A | - | *Alternaria mali* ^p^*, B. cinerea* ^p^*, Botrytis elliptica* ^p^*, Colletotrichum musae* ^p^*, C. gloeosporioides* ^c^*, F. graminearum* ^c^, *F. oxysporum* ^c^*, Glomerella cingulata* ^p^*, Podosphaera fusca* ^c^*, R. solani* ^p^*, S. cerevisae* ^p^*, S. rolfsii* ^p^ | - | Thimon *et al.*, 1992, Romero *et al.*, 2007, Hsieh *et al.*, 2008, Malfanova *et al.*, 2012, Kim *et al.*, 2010, Crane *et al.*, 2012, Gong *et al.*, 2015 |
| Lipopeptides | Iturin | Iturin C | - | *Gibberella zeae* ^p^ | - | Dunlap *et al.*, 2011 |
| Lipopeptides | Iturin | Iturin D, E | - | *B. cinerea* ^p^*, C. albicans* ^p^*, Candida tropicalis* ^p^*, M. pinodes* ^p^*, S. cerevisiae* ^p^*, S. radicinum* ^p^ | - | Besson and Michel, 1986 |
| Lipopeptides | Iturin | Mycosubtilin | *M. luteus* ^p^ | *B. cinerea* ^p^*, C. albicans* ^c^, *Cryptococcus neoformans* ^c^, *F. oxysporum* ^p^*, P. aphanidermatum* ^p^*, Pythium pastoris* ^p^*, S. cerevisiae* ^c^ | - | Thimon *et al.*, 1992, Leclère *et al.*, 2005, Fickers *et al.,* 2009 |
| Lipopeptides | Iturin | Subtulene A | *Acinetobacter calcoaceticus* ^p^*, A. tumefaciens* ^p^*, Enterobacter cloacae* ^p^*, E. coli* ^p^*, K. pneumoniae* ^p^*, Proteus mirabilis* ^p^*, P. aeruginosa* ^p^*, Pseudomonas maltophilia* ^p^*, Pseudomonas putida* ^p^*, Rhodobacter capsulatus* ^p^*, Salmonella enteritidi* ^p^ *s, Salmonella typhi* ^p^*, S. typhimurium* ^p^*, Sinorhizobium meliloti* ^p^*, Stenotrophomonas maltophilia* ^p^*, Xanthomonas campestris* ^p^ | *C. gloeosporioides* ^p^*, S. cerevisiae* ^p^*, S. rolfsii* ^p^ | - | Thasana *et al.*, 2010 |
| Lipopeptides | Surfactin | Bamylocin A | - | *B. cinerea* ^p^*, F. oxysporum* ^p^*, R. solani* ^p^ | - | Lee *et al.* 2007 |
| Lipopeptides | Surfactin | Lichenysin | *B. licheniformis* ^p^*, P. aeruginosa* ^p^*, E. coli* ^p^ | *Candida utilis* ^p^*, C. tropicalis* ^p^*, Penicilium oxalicum* ^p^*, S. cerevisiae* ^p^*, Trichoderma reesei* ^p^ | - | Jenny *et al.*, 1991 |
| Lipopeptides | Surfactin | Lichenysin A | *A. calcoaceticus* ^c^*, Alcaligenes eutrophus* ^c^*, B. subtilis* ^c^*, E. coli* ^c^*, Enterobacter* sp. ^c^, *Pseudomonas fluorescens* ^c^*, Pseudomonas proteofaciens* ^c^*, S. aureus* ^c^ | - | - | Yakimov *et al.* 1995 |
| Lipopeptides | Surfactin | Locillomycin | *X. oryzae* pv*. oryzae* ^c^*, S. aureus* ^c^ | - | Porcine Epidemic Diarrhea Virus ^c^ | Luo *et al.*, 2015 |
| Lipopeptides | Surfactin | Pumilacidin A, B, C, D, E, F, G | *Vibrio alginolyticus* ^p^*, S. aureus* ^p^ | *-* | *Herpes simplex* ^c^ | Naruse *et al.* 1990, Xiu *et al.,* 2017, Saggesse *et al.*, 2018 |
| Lipopeptides | Surfactin | Surfactin | *L. pneumophila* ^p^*, L. monocytogenes* ^p^*, P. syringae* ^c^*, R. solanacearum* ^p^*, S. aureus* ^p^, *Xanthomonas axonopodis pv. glycines* ^p^ | *A. niger* ^p^*, B. cinerea* ^p^*, F. oxysporum* ^p^*, F. solani* ^p^*, Monilia fructigena* ^p^*, Pennicilium expansum* ^p^*, P. italicum* ^p^*, R. solani ^p^* | - | Bais *et al.*, 2004, Cawoy *et al.*, 2014, Preecha *et al.*, 2010, Dimkic *et al.*, 2013, Gao L. *et al.,*  2017, Romano *et al.*, 2013, Sabaté and Audisio, 2013, Kwon and Kim, 2014, Loiseau *et al.*, 2015, Luo *et al.,* 2014 |
| Lipopeptides | Surfactin | WH1 fungin | - | *R. solani ^p^* | - | Qi *et al.*, 2010 |
| Other NRPs | Dipeptide | Bacilysin | *C. michiganense subsp. sepedonicum* ^p^, *E. amylovora* ^c^*, E. coli* ^p^.*, S. typhi* ^p^*, S. aureus* ^p^*, S. pyogenes* ^p^ | *C. albicans* ^p^*, Microcystis aeruginosa* ^c^*, Phytophthora infestans* ^p^*, S. cerevisiae* ^p^ | - | Kenig *et al.*, 1976, Kenig and Abraham, 1976, Loeffler *et al.*, 1986, Zuber *et al.*, 1993, Chen *et al.*, 2009, Wu *et al.*, 2014, Wu *et al.*, 2015a, Caulier *et al.*, 2018 |
| Other NRPs | Dipeptide | Chlorotetain | - | *A. fumigatus* ^p^*, A. niger* ^p^*, C. albicans* ^p^*, Paecilomyces uariotii* ^p^ | - | Zuber *et al.*, 1993, Rapp *et al.* 1988, Wang *et al.*, 2015 |
| Other NRPs | Siderophores | Bacillibactin | - | *F. oxysporum* f. sp. *capsici* ^p^ | - | Yu *et al.*, 2011 |
| Other NRPs | Polypeptide | Bacitracin A, F | *B. anthracis* ^c^*, B. cereus* ^p^*, E. coli* ^p^*, S. aureus* ^p^ |  | - | Hussein and AL-Janabi*.*, 2006, Furuta *et al.*, 2018 |
| Other NRPs | Polypeptide | Mycobacillin | - | *A. niger* ^p^ | - | Majumdar and Bose, 1958 |
| Other NRPs | Dipeptide | Rhizocticin A | - | *Ascodesmis sphaerospora* ^p^*, C. albicans* ^p^*, Plicaria anthracis* ^p^*, Nematospora coryli* ^p^*, Paecilomyces variotii* ^p^*, Trichophyton erinacei* ^p^ | - | Kugler *et al.*, 1990 |

^c^ Activity of isolated compound confirmed by compound purification or mutant deletion, ^p^ putative activity of the compound contained in a broth mixture.

* Two NRPs classes are reported in this review: the lipopeptides and the other NRPs.

** See Fig. 2 for the NRPs subclasses.

*** - = no activity known.

**Table S3:** VOCs produced by strains of the *B. subtilis* group.

| **VOCs class*** | **VOCs subclass**** | **Compound** | **Antimicrobial activity***** | | **References** |
| --- | --- | --- | --- | --- | --- |
|  |  |  | **Antifungal activity** | **Antibacterial activity** |  |
| Fatty acids and derivatives | Acids | Butanoic acid, 3-methyl | *F. oxysporum f. sp. lactucae* ^c^*, Moniliophthora perniciosa* ^c^ | - | Chaves-Lopez *et al.*, 2015 |
| Fatty acids and derivatives | Acids | Gentisic acid | *Colletotrichum gloeosporioides* ^c^ | - | Zheng *et al.*, 2013 |
| Fatty acids and derivatives | Acids | n-Hexanoic acid | - | *R. solanacearum* ^c^ | Raza *et al.*, 2016 |
| Fatty acids and derivatives | Acids | n-Hexadecanoic acid | *C. gloeosporioides* ^c^ | - | Zheng *et al.*, 2013 |
|  |  |  | *Alternaria brassicae* ^p^*, Alternaria solani* ^p^*, Ascochyta citrullina* ^p^*, B. cinerea* ^p^*, Cercospora kikuchii* ^p^*, Fusarium graminerum* ^p^*, F. oxysporum* ^p^*, Phoma arachnidicola* ^p^*, Rhizoctonia solani* ^p^*, Sclerotinia sclerotiorum* ^p^*, Verticillium dahiae* ^p^ | - | Liu *et al.*, 2008 |
| Fatty acids and derivatives | Acids | Octadecanoic acid, Propanoic acid, 4-hexen-1-yl ester | *A. brassicae* ^p^*, A. solani* ^p^*, A. citrullina* ^p^*, B. cinerea* ^p^*, C. kikuchii* ^p^*, F. graminerum* ^p^*, F. oxysporum* ^p^*, P. arachnidicola* ^p^*, R. solani* ^p^*, S. sclerotiorum* ^p^*, V. dahiae* ^p^ | - | Liu *et al.*, 2008 |
| Fatty acids and derivatives | Acids | Oleic acid |  | *R. solanacearum* ^c^ | Raza *et al.*, 2016 |
|  |  |  | *A. brassica* ^p^ *e, A. solani* ^p^*, A. citrullina* ^p^*, B. cinerea* ^p^*, C. kikuchii* ^p^*, F. graminerum* ^p^*, F. oxysporum* ^p^*, P. arachnidicola* ^p^*, R. solani* ^p^*, S. sclerotiorum* ^p^*, V. dahiae* ^p^ | - | Liu *et al.*, 2008 |
| Fatty acids and derivatives | Acids | Propanoic acid, 2-methyl | *F. oxysporum f. sp. lactucae* ^c^*, M. perniciosa* ^c^ | - | Chaves-Lopez *et al.*, 2015 |
| Fatty acids and derivatives | Alcohols | 3,4-dimethyl-5-hexen-3-ol, Heptanol | *A. brassica* ^p^ *e, A. solani* ^p^*, A. citrullina* ^p^*, B. cinerea* ^p^*, C. kikuchii* ^p^*, F. graminerum* ^p^*, F. oxysporum* ^p^*, P. arachnidicola* ^p^*, R. solani* ^p^*, S. sclerotiorum* ^p^*, V. dahiae* ^p^ | - | Liu *et al.*, 2008 |
| Fatty acids and derivatives | Alcohols | 2-Undecanol | *F. oxysporum f. sp. cubense* ^c^ | - | Yuan *et al.*, 2012b |
| Fatty acids and derivatives | Alcohols | 1-Butanol | *F. oxysporum f. sp. lactucae* ^c^*, M. perniciosa* ^c^ | - | Chaves-Lopez *et al.*, 2015 |
| Fatty acids and derivatives | Alcohols | 1-Butanol, 3-methyl- | *A. brassicae, A. solani, A. citrullina, B. cinerea, C. kikuchii, F. graminerum, F. oxysporum, P. arachnidicola, R. solani, S. sclerotiorum, V. dahiae,* | - | Liu *et al.*, 2008 |
|  |  |  | *F. oxysporum f. sp. lactucae* ^c^*, M. perniciosa* ^c^ | - | Chaves-Lopez *et al.*, 2015 |
| Fatty acids and derivatives | Alcohols | Hexanol,2-ethyl | *B. cinerea* ^p^ | - | Chen *et al.*, 2008 |
|  |  |  | *F. oxysporum f. sp. cubense* ^c^ | - | Yuan *et al.*, 2012b |
| Fatty acids and derivatives | Aldehydes | 2,4-Heptadienal, (E,E)-, 2-Decenal, (E)-, 2-Heptenal, (Z)-, 2-Nonenal, (E)-, 2-Octenal, (E)-, 2-Undecenal, 2,4 Decadienal, Octanal | *A. brassica* ^p^ *e, A. solani* ^p^*, A. citrullina* ^p^*, B. cinerea* ^p^*, C. kikuchii* ^p^*, F. graminerum* ^p^*, F. oxysporum* ^p^*, P. arachnidicola* ^p^*, R. solani* ^p^*, S. sclerotiorum* ^p^*, V. dahiae* ^p^ | - | Liu *et al.*, 2008 |
|  |  | Decanal | *F. oxysporum f. sp. cubense* ^c^ | - | Yuan *et al.*, 2012b |
| Fatty acids and derivatives | Aldehydes | Nonanal | *A. brassicae, A. solani, A. citrullina, B. cinerea, C. kikuchii, F. graminerum, F. oxysporum, P. arachnidicola, R. solani, S. sclerotiorum, V. dahiae,* | - | Liu *et al.*, 2008, Yuan *et al.*, 2012b, Wang *et al.*, 2013 |
|  |  |  | - | *R. solanacearum* ^c^ | Raza *et al.*, 2016 |
| Fatty acids and derivatives | Alkanes | Eicosane, 10-methyl- | *B. cinerea* ^p^ | - | Chen *et al.*, 2008 |
| Fatty acids and derivatives | Alkanes | Heneicosane | *C. gloeosporioides* ^c^ | - | Zheng *et al.*, 2013 |
| Fatty acids and derivatives | Alkanes | Heptadecane | - | *R. solanacearum* ^c^ | Raza *et al.*, 2016 |
| Fatty acids and derivatives | Alkanes | Heptadecane, 2,6,10,15-tetramethyl- | *B. cinerea* ^p^ | - | Chen *et al.*, 2008 |
| Fatty acids and derivatives | Alkanes | Heptane, 2-methyl-7-oxabicyclo[2.2.1] | *A. brassica* ^p^ *e, A. solani* ^p^*, A. citrullina* ^p^*, B. cinerea* ^p^*, C. kikuchii* ^p^*, F. graminerum* ^p^*, F. oxysporum* ^p^*, P. arachnidicola* ^p^*, R. solani* ^p^*, S. sclerotiorum* ^p^*, V. dahiae* ^p^ | - | Liu *et al.*, 2008 |
|  |  |  | *F. oxysporum f. sp. cubense* ^c^ | - | Wang *et al.*, 2013 |
| Fatty acids and derivatives | Alkanes | Hexadecane, 2,6,11,15-tetramethyl- | *B. cinerea* ^p^ | - | Chen *et al.*, 2008 |
| Fatty acids and derivatives | Alkanes | Nonadecane, 9-methyl- | *B. cinerea* ^p^ | - | Chen *et al.*, 2008 |
| Fatty acids and derivatives | Alkanes | Nonadecane,10-methyl- | *B. cinerea* ^p^ | - | Chen *et al.*, 2008 |
| Fatty acids and derivatives | Alkanes | Pentadecane | *F. oxysporum f. sp. cubense* ^c^ | - | Wang *et al.*, 2013 |
| Fatty acids and derivatives | Alkanes | Pentadecane, 8-hexyl- | *B. cinerea* ^p^ | - | Chen *et al.*, 2008 |
| Fatty acids and derivatives | Alkanes | Tetradecane | *F. oxysporum f. sp. cubense* ^c^ | - | Wang *et al.*, 2013 |
| Fatty acids and derivatives | Alkanes | Tetradecane, 2,6,10-trimethyl- | *B. cinerea* ^p^ | - | Chen *et al.*, 2008 |
| Fatty acids and derivatives | Alkanes | Undecane,1,2-methyl | *F. oxysporum f. sp. cubense* ^c^ | - | Wang *et al.*, 2013 |
| Fatty acids and derivatives | Alkenes | 1H-indene, 1-methylene- | *A. brassica* ^p^ *e, A. solani* ^p^*, A. citrullina* ^p^*, B. cinerea* ^p^*, C. kikuchii* ^p^*, F. graminerum* ^p^*, F. oxysporum* ^p^*, P. arachnidicola* ^p^*, R. solani* ^p^*, S. sclerotiorum* ^p^*, V. dahiae* ^p^ | - | Liu *et al.*, 2008 |
| Fatty acids and derivatives | Alkenes | 1,3-butadiene | - | *R. solanacearum* ^c^ | Tahir *et al.*, 2017 |
| Fatty acids and derivatives | Alkenes | 1-decene, 8-methyl- | *A. brassica* ^p^ *e, A. solani* ^p^*, A. citrullina* ^p^*, B. cinerea* ^p^*, C. kikuchii* ^p^*, F. graminerum* ^p^*, F. oxysporum* ^p^*, P. arachnidicola* ^p^*, R. solani* ^p^*, S. sclerotiorum* ^p^*, V. dahiae* ^p^ | - | Liu *et al.*, 2008 |
| Fatty acids and derivatives | Alkenes | 1,3- pentadiene | *B. cinerea* ^c^ | - | Gotor-Vila *et al.*, 2017 |
| Fatty acids and derivatives | Benzenoids | β-Benzeneethanamine | *C. gloeosporioides* ^c^ | - | Zheng *et al.*, 2013 |
| Fatty acids and derivatives | Benzenoids | 1,2-Benzisothiazol-3(2H)-one | - | *R. solanacearum* ^c^ | Tahir *et al.*, 2017 |
| Fatty acids and derivatives | Benzenoids | 2,4-bis(2-Methylpropyl)-phenol | *B. cinerea* ^p^ | - | Chen *et al.*, 2008 |
| Fatty acids and derivatives | Benzenoids | 2-Phenylethanol | - | *R. solanacearum* ^c^ | Raza *et al.*, 2016 |
| Fatty acids and derivatives | Benzenoids | 4-Hydroxybenzaldehyde | *B. cinerea* ^p^ | - | Chen *et al.*, 2008 |
|  |  |  | *F. oxysporum f. sp. cubense* ^c^ | - | Wang *et al.*, 2013 |
|  |  |  | - | *R. solanacearum* ^c^ | Tahir *et al.*, 2017 |
| Fatty acids and derivatives | Benzenoids | Benzene, 1,2,4,5-tetramethyl | *B. cinerea* ^p^ | - | Chen *et al.*, 2008 |
| Fatty acids and derivatives | Benzenoids | Benzene, 1,2,4-trimethyl | *F. oxysporum f. sp. cubense* ^c^ | - | Yuan *et al.*, 2012b |
| Fatty acids and derivatives | Benzenoids | Benzene, 1-methyl-4-(1-methylethyl)- | *F. oxysporum f. sp. cubense* ^c^ | - | Yuan *et al.*, 2012b |
| Fatty acids and derivatives | Benzenoids | Benzene, 2-propenyl | *F. oxysporum f. sp. cubense* ^c^ | - | Wang *et al.*, 2013 |
| Fatty acids and derivatives | Benzenoids | Benzene,1,4-dichloro | *F. oxysporum f. sp. cubense* ^c^ | - | Wang *et al.*, 2013 |
| Fatty acids and derivatives | Benzenoids | Benzothiazole | *F. oxysporum f. sp. cubense* ^c^ | - | Yuan *et al.*, 2012b |
|  |  |  | *A. solani* ^c^*, B. cinerea* ^c^ | - | Gao Z. *et al.*, 2017 |
| Fatty acids and derivatives | Benzenoids | Butylated hydroxytoluene | *C. gloeosporioides* ^c^ | - | Zheng *et al.*, 2013 |
| Fatty acids and derivatives | Benzenoids | Ethylbenzene | *F. oxysporum f. sp. cubense* ^c^ | - | Yuan *et al.*, 2012b |
|  |  |  | *F. oxysporum f. sp. cubense* ^c^ | - | Yuan *et al.*, 2012b |
| Fatty acids and derivatives | Benzenoids | Phenol, 2,4-bis(1,1- dimethylethyl) | *A. solani* ^c^*, B. cinerea* ^c^ | - | Gao Z. *et al.*, 2017 |
| Fatty acids and derivatives | Benzenoids | Phenol, 4,4′-(1-methylethylidene) bis- | - | *R. solanacearum* ^c^ | Raza *et al.*, 2016 |
| Fatty acids and derivatives | Benzenoids | Phenol, 4-chloro-3-methyl | *A. solani* ^c^*, B. cinerea* ^c^ | - | Gao Z. *et al.*, 2017 |
| Fatty acids and derivatives | Benzenoids | Phenol,2,3,6-trimethyl- | *F. oxysporum f. sp. cubense* ^c^ | - | Yuan *et al.*, 2012b |
| Fatty acids and derivatives | Benzenoids | P-xylene | *F. oxysporum f. sp. cubense* ^c^ | - | Yuan *et al.*, 2012b |
|  |  |  | *F. oxysporum f. sp. cubense* ^c^ | - | Wang *et al.*, 2013 |
| Fatty acids and derivatives | Benzenoids | Styrene | *F. oxysporum f. sp. cubense* ^c^ | - | Yuan *et al.*, 2012b |
| Fatty acids and derivatives | Benzenoids | Toluene | *F. oxysporum f. sp. cubense* ^c^ | - | Yuan *et al.*, 2012b |
| Fatty acids and derivatives | Esters | Ethyl acetate | *F. oxysporum f. sp. lactucae* ^c^*, M. perniciosa* ^c^ | - | Chaves-Lopez *et al.*, 2015 |
| Fatty acids and derivatives | Furans | Furan, 2-pentyl- | *A. brassica* ^p^ *e, A. solani* ^p^*, A. citrullina* ^p^*, B. cinerea* ^p^*, C. kikuchii* ^p^*, F. graminerum* ^p^*, F. oxysporum* ^p^*, P. arachnidicola* ^p^*, R. solani* ^p^*, S. sclerotiorum* ^p^*, V. dahiae* ^p^ | - | Liu *et al.*, 2008 |
| Fatty acids and derivatives | Ketones | Acetoin | *Penicillium crustosum* ^c^ | - | Arrebola *et al.*, 2010 |
|  |  |  | *B. cinerea* ^c^ | - | Gotor-Vila *et al.*, 2017 |
| Fatty acids and derivatives | Ketones | Butan-2-one | *F. oxysporum f. sp. lactucae* ^c^*, M. perniciosa* ^c^ | - | Chaves-Lopez *et al.*, 2015 |
| Fatty acids and derivatives | Ketones | Butanone, 3-hydroxy-2- | *F. oxysporum f. sp. lactucae* ^c^*, M. perniciosa* ^c^ | - | Chaves-Lopez *et al.*, 2015 |
|  |  |  | *C. gloeosporioides* ^c^ | - | Zheng *et al.*, 2013 |
|  |  |  | *F. oxysporum f. sp. cubense* ^c^ | - | Wang *et al.*, 2013 |
|  |  |  | *F. oxysporum f. sp. cubense* ^c^ | - | Yuan *et al.*, 2012b |
| Fatty acids and derivatives | Ketones | Dodecan-2-one | *F. oxysporum f. sp. cubense* ^c^ | - | Wang *et al.*, 2013 |
| Fatty acids and derivatives | Ketones | Ethanone, 1-(4-methylphenyl) | *F. oxysporum f. sp. cubense* ^c^ | - | Wang *et al.*, 2013 |
|  |  |  | *B. cinerea* ^p^ | - | Wang *et al.*, 2013 |
|  |  |  | *F. oxysporum f. sp. cubense* ^c^ | - | Chen *et al.*, 2008 |
|  |  |  | *C. gloeosporioides* ^c^ | - | Yuan *et al.*, 2012b |
|  |  |  | - | *R. solanacearum* ^c^ | Zheng *et al.*, 2013 |
|  |  |  | *A. brassica* ^p^ *e, A. solani* ^p^*, A. citrullina* ^p^*, B. cinerea* ^p^*, C. kikuchii* ^p^*, F. graminerum* ^p^*, F. oxysporum* ^p^*, P. arachnidicola* ^p^*, R. solani* ^p^*, S. sclerotiorum* ^p^*, V. dahiae* ^p^ | - | Raza *et al.*, 2016 |
|  |  |  | *F. oxysporum f. sp. lactucae* ^c^*, M. perniciosa* ^c^ | - | Wang *et al.*, 2013 |
| Fatty acids and derivatives | Ketones | Pentanone, 2,2,4-trimethyl-3- | - | *R. solanacearum* ^c^ | Liu *et al.*, 2008 |
| Fatty acids and derivatives | Ketones | Propan-2-one | *F. oxysporum f. sp. cubense* ^c^ | - | Chaves-Lopez *et al.*, 2015 |
| Fatty acids and derivatives | Ketones | Tridecan-2-one | *F. oxysporum f. sp. cubense* ^c^ | - | Raza *et al.*, 2016 |
|  |  |  | *A. brassica* ^p^ *e, A. solani* ^p^*, A. citrullina* ^p^*, B. cinerea* ^p^*, C. kikuchii* ^p^*, F. graminerum* ^p^*, F. oxysporum* ^p^*, P. arachnidicola* ^p^*, R. solani* ^p^*, S. sclerotiorum* ^p^*, V. dahiae* ^p^ | - | Wang *et al.*, 2013 |
|  |  |  | *B. cinerea* ^p^ | - | Yuan *et al.*, 2012b |
|  |  |  | *Phaeomoniella chlamydospora* ^c^ | - | Raza *et al.*, 2016 |
|  |  |  | *A. solani* ^c^ | - | Wang *et al.*, 2013 |
| Nitrogen-containing | Azoles | 1H-imidazole, 1-ethyl | *F. oxysporum f. sp. lactucae* ^c^*, M. perniciosa* ^c^ | - | Liu *et al.*, 2008 |
| Nitrogen-containing |  | Ammonium acetate | *B. cinerea* ^p^ | - | Chen *et al.*, 2008 |
| Nitrogen-containing | Pyrazines | Pyrazine, 2,5-dimethyl | *F. oxysporum f. sp. lactucae* ^c^*, M. perniciosa* ^c^ | - | Haidar *et al.*, 2016 |
|  |  |  | *F. oxysporum f. sp. lactucae* ^c^*, M. perniciosa* ^c^ | - | Gao Z. *et al.*, 2017 |
|  |  |  | *S. sclerotiorum* ^c^ | *-* |  |
| Nitrogen-containing | Pyrazines | Pyrazine, 2-ethyl-3,5-dimethyl | *B. cinera* ^c^*, Monilia fructicola* ^c^*, Monilinia laxa* ^c^ | - | Chaves-Lopez *et al.*, 2015 |
| Nitrogen-containing | Pyrazines | Pyrazine, tetramethyl- | *F. oxysporum f. sp. lactucae* ^c^*, Moniliophthora perniciosa* ^c^ | - | Chen *et al.*, 2008 |
| Nitrogen-containing | Pyrazines | Pyrazine,2,3,5,6-tetramethyl | *Colletotrichum gloeosporioides* ^c^ | - | Chaves-Lopez *et al.*, 2015 |
| Sulphur-containing | - | Carbon disulphide | - | *R. solanacearum* ^c^ | Chaves-Lopez *et al.*, 2015 |
| Sulphur-containing | - | Dimethyl trisulfide | *C. gloeosporioides* ^c^ | - | Giorgio *et al.*, 2015 |
| Sulphur-containing | - | Thiophene | *Alternaria brassicae* ^p^*, Alternaria solani* ^p^*, Ascochyta citrullina* ^p^*, B. cinerea* ^p^*, Cercospora kikuchii* ^p^*, Fusarium graminerum* ^p^*, F. oxysporum* ^p^*, Phoma arachnidicola* ^p^*, Rhizoctonia solani* ^p^*, Sclerotinia sclerotiorum* ^p^*, Verticillium dahiae* ^p^ | - | Gotor-Vila *et al.*, 2017 |

^c^ Activity of isolated compound, ^p^ putative activity of the compound contained in a broad mixture.

* Three VOCs classes are secreted by *B. subtilis* strain: the fatty acids and derivatives, the nitrogen-containing as well as the sulphur-containing compounds.

** See Fig. 2 for the VOCs subclasses.

*** - = no activity known.
