# Supplementary figures and images for: Overview of the Antimicrobial Compounds Produced by Members of the Bacillus subtilis Group
Source: Front Microbiol. 2019 Feb 26;10:302. doi: 10.3389/fmicb.2019.00302 (PMC6401651; doi:10.3389/fmicb.2019.00302)

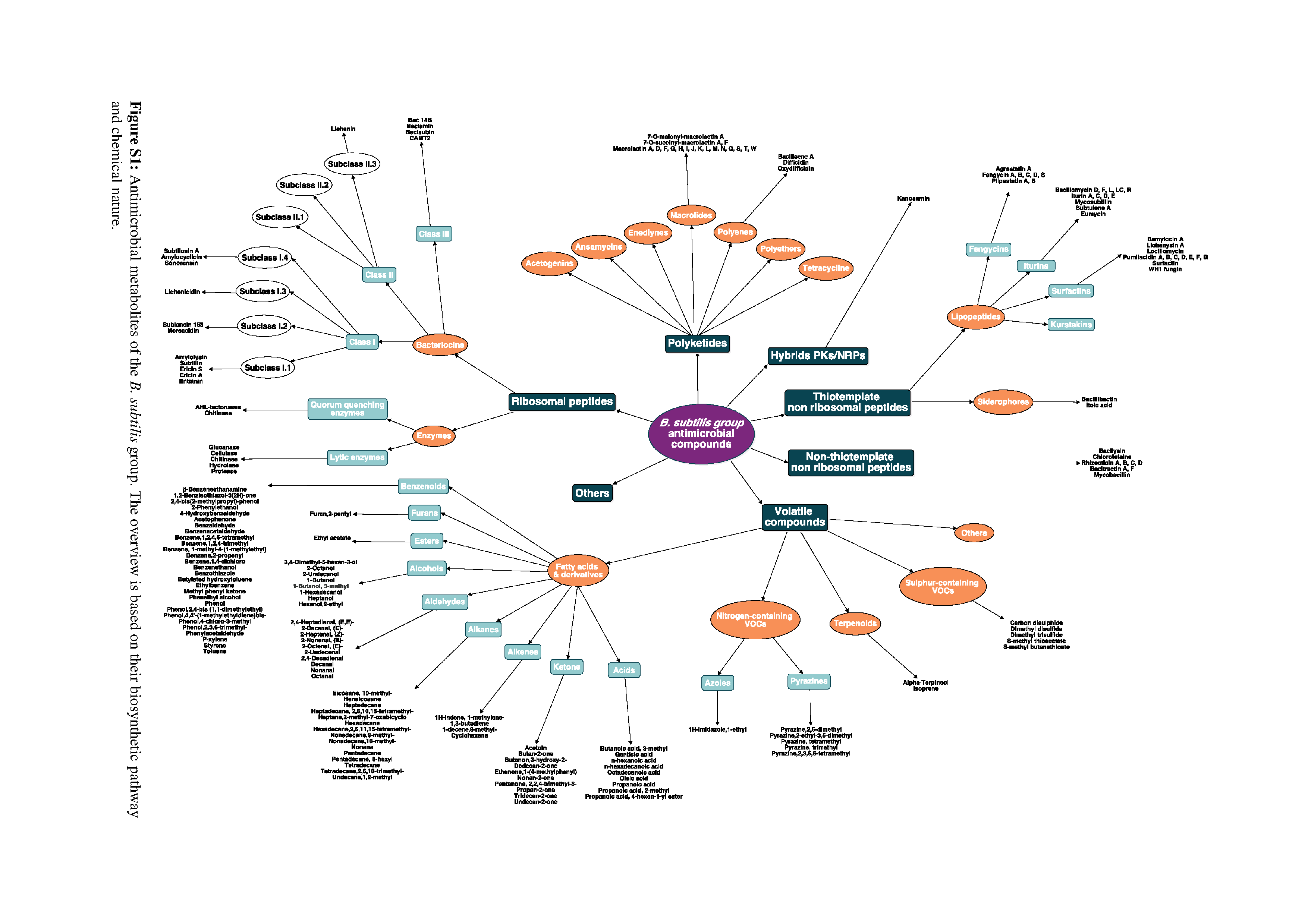

Supplement: Supplementary file 2 [file Image_1.tif]
